# Wine Volatilome as Affected by Tartaric Stabilization Treatments: Cold Stabilization, Carboxymethylcellulose and Metatartaric Acid

**DOI:** 10.3390/foods13172734

**Published:** 2024-08-28

**Authors:** Fernanda Cosme, Rui Oliveira, Luís Filipe-Ribeiro, Fernando M. Nunes

**Affiliations:** 1 CQ-VR, Chemistry Research Centre—Vila Real, Food and Wine Chemistry Lab, Biology and Environment Department, University of Trás-os-Montes and Alto Douro, 5000-801 Vila Real, Portugal; 2 CQ-VR, Chemistry Research Centre—Vila Real, Food and Wine Chemistry Lab, University of Trás-os-Montes and Alto Douro, 5000-801 Vila Real, Portugal; ruioliveirapf@gmail.com (R.O.); fmota@utad.pt (L.F.-R.); 3 CQ-VR, Chemistry Research Centre—Vila Real, Food and Wine Chemistry Lab, Chemistry Department, University of Trás-os-Montes and Alto Douro, 5000-801 Vila Real, Portugal

**Keywords:** wine, tartaric stability, cold stabilization, sodium carboxymethylcellulose, metatartaric acid, volatilome, phenolic composition, chromatic characteristics

## Abstract

The primary cause of bottled wine sediment is tartrate crystal precipitation. To prevent this, wines undergo a stabilization process before bottling. The most commonly used method is cold stabilization, which induces the precipitation of tartrate crystals that are then removed, thereby eliminating the excess ions that cause instability in wine. Another approach to tartaric stabilization is using enological stabilizers with a colloid protective effect, which prevents the formation of tartrate crystals. The most commonly used tartaric stabilizers are sodium carboxymethylcellulose (CMC) and metatartaric acid. However, both have drawbacks: they are semi-synthetic products, and metatartaric acid degrades over time, losing its stabilizing effect. This study aims to compare the effects of cold stabilization, stabilization with CMC, and metatartaric acid on the chemical composition, particularly the volatilome, of white, rosé, and red wines. Cold stabilization significantly impacted the wine volatilome, especially in white and rosé wines, by decreasing total alcohols and increasing total esters. It also reduced the color intensity of rosé and red wines by lowering monomeric anthocyanins. In contrast, enological stabilizers had minimal impact on the wines’ phenolic composition, chromatic characteristics, and volatilome. The sensory impact of cold stabilization is complex; it can potentially enhance the aroma of white and rosé wines by increasing ester VOCs and decreasing higher alcohols, but it negatively affects the color of rosé and red wines.

## 1. Introduction

During the winemaking and aging process, potassium bitartrate (KHT) and, to a lesser extent, calcium tartrate (CaT) crystals can precipitate, especially when the grape must or wine is exposed to low temperatures [1]. Even after bottling, some wines can develop tartaric crystal sediments. Although these precipitations are natural and harmless, they negatively impact consumers, who often associate them with defects such as microbiological issues, sugar crystals, chemical additives, or glass splinters, leading to rejected purchases [2,3,4]. Therefore, stabilizing wines against tartaric precipitations is necessary. Current methods include subtractive techniques, which remove potassium and calcium ions (cold stabilization, electrodialysis, and ion exchange resins), and additive methods, which involve adding protective colloids (metatartaric acid, sodium carboxymethylcellulose, mannoproteins, and potassium polyaspartate) to prevent or delay crystallization [5].

The most traditional method of tartaric stabilization is cold treatment, which involves cooling the wine to near its freezing point and storing it in isothermal vats or refrigeration chambers for 3–4 days to 3–4 weeks, with 1 week being the most common duration. Its effectiveness depends on the wine composition [6]. Low temperatures induce the formation of potassium bitartrate crystals, but this method is both time and energy-consuming [6,7]. The treatment duration varies: red wines take 2 to 3 weeks because of the presence of protective colloids, while white and rosé wines require 7 to 12 days [4]. Higher energy costs are associated with the cooling equipment, and the prolonged low temperatures increase oxygen dissolution (approximately 11 mg/L at 0 °C and 8 mg/L at 15 °C), leading to oxidation [8] and the potential loss of color [8], aroma compounds, and polysaccharides [9]. Cold treatment is expensive and environmentally impactful because of its high water and energy consumption [1,10]. Studies show that alternative methods, such as metatartaric acid and sodium carboxymethylcellulose (CMC), are more cost-effective [1]. The environmental impact of cold treatment includes significant water consumption (1.54 to 1.88 L/hL of wine for processing and cleaning), waste production (organic load and composition of wastewater and residues), and high energy usage (1 to 1.7 kWh/hL of wine) [1,10,11,12,13]. Effluents from cold stabilization reveal that 66.6% of the chemical oxygen demand (COD) comes from diatomaceous earth, 21.8% from the washing of the filter, and 11.4% from washing the cold treatment tank [10]. Reducing the environmental impacts requires eco-friendly stabilization processes and better resource management. Winery wastewater volumes vary depending on the winery, ranging from 0.5 to 10 L/L of wine, with an average value of around 1 L/L of wine [14].

The addition of enological stabilizers with protective colloid properties, approved by the OIV, prevents tartaric instability in wine. It is a simple and low cost operation [15]. CMC, a cellulose derivative, was approved for enological use by the OIV in 2009, with an updated limit of 200 mg/L for white and rosé wine as of 2023 [5]. CMC is obtained by the etherification of the free primary alcohol groups of glucopyranose units through glycosidic bonds. It is characterized by the degree of substitution (DS) and degree of polymerization (DP), with DS representing the ratio of glucose units replaced with carboxymethyl groups [16,17] and DP representing the average number of glucose units per polymeric chain [17,18]. For enological use, the DS must be between 0.60 and 0.95 and the DP between 80 and 1.500 [5].

CMC carries a negative charge at wine pH, allowing it to adsorb KHT crystals and inhibit their growth, as well as interact with K^+^ and Ca^2+^ ions, reducing the free ions involved in crystallization [19]. It also changes the shape of the KHT crystals, delaying their formation [20]. A higher DS increases CMC effectiveness by providing more cation adsorption sites [17,21]. According to Salagoїty et al. [22], CMC does not alter sensory characteristics at the maximum OIV dose. Even at fifteen times the maximum authorized dose, no impact on taste or odor was detected. CMC is economically viable, easy to use, and inert [16], remaining stable for over 12 months even at high temperatures [20]. Bowyer et al. [23] observed no pH change compared with metatartaric acid application.

CMC use is currently limited to white, rosé, and sparkling wines [5] because of its reduced efficiency in red wine [24] and the potential for color loss and turbidity [1]. The color loss in red wine may be associated with proteins involved in turbidity formation [25] or interaction with phenolic compounds [24]. Claus et al. [25] found that CMC can prevent bitartrate crystal growth in red wines, though some wines showed protein turbidity. Sommer et al. [26] demonstrated that color loss associated with CMC required proteins, likely due to anthocyanin precipitation via protein bridging. Thus, CMC effectively increases tartaric stability in red wine without impacting phenolic composition, sensory characteristics, or the stability of coloring matter [27,28].

Metatartaric acid, a polyester derived from the esterification of tartaric acid, is produced under controlled high temperatures (150 to 160 °C in a vacuum). This process involves intramolecular dehydration and esterification, resulting in a polymeric, yellowish–brown mass without a defined chemical structure and an esterification index of 30 to 40% [8,29]. It may contain impurities such as pyruvic acid, which can represent 1 to 6% of the metatartaric acid weight, depending on the production conditions. The use of metatartaric acid in wine is permitted at a maximum dose of 100 mg/L (E353) [5].

The application of metatartaric acid provides a short- to medium-term protective effect, depending on the storage temperature, which is often sufficient for the typically short turnover rates of white wines [29]. Metatartaric acid delays the crystal growth of potassium bitartrate and calcium tartrate, preventing their precipitation. It is highly soluble in water and alcohol, and while it hydrolyzes quickly in an aqueous solution at 100 °C, it does so more slowly at lower temperatures, which helps to maintain its protective effect [8]. Therefore, after its application, it is essential to ensure maintenance and storage at low temperatures.

Volatile organic compounds (VOCs) play a crucial role in the development of food odors, a key factor in food acceptability [30]. In wine, volatile compounds significantly contribute to its aroma and flavor, making volatilomics, the study of these compounds, essential for assessing wine quality. By analyzing the volatile profile, winemakers can evaluate and control the aroma intensity, complexity, balance, and overall character, thus influencing the impact of grape variety, terroir, fermentation, aging, and storage. This knowledge enables winemakers to make informed decisions throughout the winemaking process to enhance or maintain the desired sensory characteristics.

Headspace solid-phase microextraction (HS-SPME) is a state-of-the-art technique that is simple, effective, and cost-efficient for analyzing aroma compounds, as it requires minimal solvent manipulation [31,32,33,34]. Volatilomics helps winemakers optimize production practices, preserve wine quality, and meet consumer preferences, thereby enhancing the sensory experience and overall enjoyment of wines.

Although articles compare cold treatment with electrodialysis [12,35] and different protective colloids [3,17,36], there is no comprehensive comparison between cold treatment and protective colloid stabilization. Therefore, the main objective of this work is to establish a detailed comparison between wines stabilized by cold treatments and the same wines stabilized with CMC and metatartaric acid. This study will include an in-depth analysis of the wine’s chemical composition (tartaric acid, metallic, phenolics, aroma, and color characterization) as well as a characterization of the wine’s volatilome.

## 2. Materials and Methods

### 2.1. Wine Samples

The wines used in this work were produced at Quinta D’Amares, located in Braga—Portugal, and are classified as DOC “Vinho Verde.” These include a monovarietal white wine from the Loureiro grape variety, a monovarietal rosé wine from the Padeiro de Basto grape variety, and a red wine blend composed of 90% Vinhão and 10% Touriga Nacional. The white wine was produced through fermentation at 14 °C, followed by aging on fine lees with periodic pumping over with nitrogen (battonage). The wine was then fined with gelatine, PVPP, and bentonite to remove undesirable phenolic and protein compounds before being filtered. The rosé wine underwent a similar process, differing only in that its fermentation was performed at 15 °C. The red wine was fermented in a rotary fermentation tank at 20 °C, followed by complete malolactic fermentation. It was then fined with gelatine to reduce astringency by decreasing tannins and subsequently filtered. The physicochemical characteristics of the white, rosé, and red wines are shown in Table 1.

### 2.2. Tartaric Stabilization Experiments

Part of the wine was stabilized on an industrial scale using cold treatment at a temperature of −4 °C for 6 days. The other stabilization treatments were carried out with CMC and metatartaric acid at a concentration of 100 mg/L. Each enological product was prepared according to the manufacturer’s specifications. Wine without any treatment was used as a control. The experiments were performed in duplicate in 500 mL graduated cylinders and allowed to remain in contact with the unstable tartaric wine for 7 days at 20 °C. After that, the wine was bottled for chemical analysis. Samples were centrifuged at 537.6 g for 15 min before analysis. All the assays were repeated twice, and all analyses were performed in duplicate.

### 2.3. Mini Contact Test

The variation in electrical conductivity (Δx) of the wine was measured by placing the wine at 0 °C under continuous stirring for 15 min after the addition of micronized potassium bitartrate crystals. This mini-contact test was carried out using a Tartar Check (Ing. C. Bullio, San Prospero, Italy). The variation in electrical conductivity is expressed in μS/cm and indicates the level of stability. For red wine, the stability levels are as follows (Δx): <40 very stable, 40–60 stable, 60–80 warning, and >80 not stable. For white and rosé wine, the stability levels are (Δx) < 30 very stable, 30–50 stable, 50–70 warning, and >70 not stable [37].

### 2.4. Conventional Enological Parameters and Mineral Composition

Volatile acidity, alcohol strengths (%, *v*/*v*), titratable acidity, pH, and mineral composition (potassium, calcium, sodium, magnesium, and iron) were determined according to Organisation International de la Vigne et du Vin methods [38].

### 2.5. Tartaric Acid Determination

Colorimetric determination of tartaric acid was performed using the Rebelein method, as modified by Vidal and Blouin [39].

### 2.6. Quantification of Flavonoids, Non-Flavonoids and Total Phenols

The phenolic content of the wines was quantified using the absorbance at 280 nm before and after the precipitation of flavonoid phenols through a reaction with formaldehyde, according to the method by Kramling and Singleton [40]. Non-flavonoid phenolic compounds were also quantified following Kramling and Singleton [40]. Total phenolic compounds were determined using a spectrophotometric method as described by Ribéreau-Gayon et al. [8]. Flavonoid phenolic compounds were calculated as the difference between total phenolic compounds and non-flavonoid phenolic compounds [40]. Quantifications were performed using a gallic acid calibration curve, and the results were expressed as gallic acid equivalents per liter (GAE/L). All analyses were conducted in duplicate.

### 2.7. Color, Chromatic Characterization, and Pigments

The color intensity was determined based on the sum of the absorbance at 620, 520, and 420 nm (1 mm cell), and the hue was determined as the ratio of absorbance at 420 and 520 nm [38]. The color of white wine was measured based on the absorbance at 420 nm (10 mm cell) according to the OIV guidelines [38]. The absorption spectra of wine samples were scanned over the range of 380–770 nm using quartz cells. Data were collected to determine L* (lightness), a* (redness), and b* (yellowness) coordinates using the CIELab method [38]. The Chroma [C* = [(a*)^2^ + (b*)^2^]^1/2^] and hue-angle [h^o^ = tang^_1^(b*/a*)] values were also calculated. The content of total and colored anthocyanins, as well as total and polymeric pigments, was determined using the method proposed by Somers and Evans [41].

### 2.8. Test for Browning Potential

Test tubes were filled with 20 mL of the wine to be tested. Control and test samples were thoroughly sparged with nitrogen and oxygen, respectively. All tubes were hermetically sealed and maintained at 55 °C for 5 days. The test was conducted on both treated and untreated wine, and the browning value difference was calculated by measuring the increase in A420 nm according to the methodology proposed by Singleton and Kramling [42].

### 2.9. HPLC-DAD Analysis of Monomeric Anthocyanins, (+)-Catechin, and Phenolic Acids

Monomeric anthocyanins, (+)-catechin, and phenolic acids were analyzed by injecting the wine into an HPLC system equipped with an Ultimate 3000 Dionex liquid chromatograph, a PDA-100 photodiode array detector, and an Ultimate 3000 Dionex pump. Separation was achieved on a C18 column (250 mm × 4.5 mm, 5 μm particle size, ACE, Scotland) with a flow rate of 1 mL/min at 35 °C. The analysis conditions utilized 5% aqueous formic acid (A) and methanol (B), with the following solvent gradient: 5% B from 0 to 5 min, followed by a linear gradient up to 65% B until 65 min, and from 65 to 67 min down to 5% B. The injection volume was 50 μL, and detection was performed from 200 to 650 nm with a run time of 75 min per sample [17].

### 2.10. Quantification of Monomeric Anthocyanins, (+)-Catechin, and Phenolic Acids

Calibration curves using authentic standards of caffeic acid, *p*-coumaric acid, ferulic acid, gallic acid, and (+)-catechin were used to quantify phenolic acids and (+)-catechin. Results for *trans*-caftaric acid and 2-*S*-glutathionylcaftaric acid (GRP) were expressed as caffeic acid equivalents, while coutaric acid and ethylcoumaric acid were expressed as 4-hydroxycinnamic acid equivalents. For the quantification of monomeric anthocyanins, calibration curves of authentic standards for cyanidin-3-glucoside, malvidin-3-glucoside, and peonidin-3-glucoside were used. Using the molar absorptivity coefficients (ε) and extrapolation, slopes were determined for delphinidin-3-glucoside, petunidin-3-glucoside, and malvidin-3-coumaroylglucoside. Results for delphinidin-3-acetylglucoside, petunidin-3-acetylglucoside, peonidin-3-acetylglucoside, cyanidin-3-acetylglucoside, and cyanidin-3-coumaroylglucoside were expressed as their respective glucoside equivalents.

### 2.11. Determination of the Volatilome GC-MS-HS/SPME

The identification and comparative semi-quantification of volatile compounds were conducted using gas chromatography (GC) (Trace GC Ultra) with a mass spectrometer as a detection system (PolarisQ). Solid-phase microextraction (SPME) was used for extraction, performed for 40 min at 300 rpm, with the SPME held at 35 °C and the transfer zone at 250 °C. Injections were carried out by an automatic injector (AS 3000), with 5 μL injections in splitless mode at 270 °C for 2 min, using a single taper liner with a 5 mm diameter. The chromatographic column used was an OPTIMA FFAP, with a length of 30 m, a diameter of 0.32 mm, and a film thickness of 0.25 μm. Helium was used as the carrier gas at a flow rate of 1.5 mL/min. The oven temperature program was as follows: 40 °C for the first 2 min, then increasing to 220 °C at a rate of 2 °C/min, followed by a rise to 250 °C at 10 °C /min, which was maintained for 3 min. The total program duration was 98 min. The mass spectrometer operated in full-scan mode for positive ions, with a mass range from 45 to 650, and the ion source at a temperature of 220 °C [43]. Identification was based on a combination of mass spectral data from the Wiley 7 NIST 2005 mass spectral library and Kovats index values determined for most volatile compounds, which were then compared with those in the Wiley library. For calculating Kovats indices, a mixture of n-alkanes (C8–C20) supplied by Supelco was dissolved in n-hexane, and the retention time of standards was determined using the described temperature-programmed. Aroma compounds with ≥80% similarity to the Wiley mass spectral library were tentatively identified using the GC/MS spectra. For compounds with Kovats index values < 800, where a mixture of n-alkanes < C8 was not available, identification was based on mass spectral data in the Wiley 7 NIST 2005 library.

### 2.12. Statistical Treatment

Data are presented as means ± standard deviation. All physicochemical data were statistically analyzed using GraphPad Prism 7 software (GraphPad Software, Inc., San Diego, CA, USA). Analysis of variance (ANOVA) was performed, followed by Tukey’s honestly significant difference (HSD, 5% level) post hoc test for physicochemical data. These analyses were conducted using Statistica 7 Software (StatSoft, Tulsa, OK, USA).

Principal Component Analysis (PCA) is a chemometric method used for data reduction and analysis of high-dimensional data. It decomposes data into loading (volatilome of wines) and score (wine samples) matrices, with principal components capturing the total variance in uncorrelated combinations. As an unsupervised method, PCA does not require data grouping and simplifies interpretation. Varimax rotation further refines components, concentrating variance on fewer variables for clearer results [44].

## 3. Results and Discussion

### 3.1. Impact of Cold Stabilization, Carboxymethylcellulose, and Metatartaric Acid on the Wines Tartaric Stability, Tartaric Acid and Mineral Composition

The application of cold stabilization to white, rosé, and red wines resulted in a significant increase in tartaric stability, as evidenced by a significant decrease in ∆x. Martínez-Pérez et al. [4] also showed that cold treatment was effective for tartaric stabilization in red wines, maintaining its stability even after a year in the bottle. A similar trend was observed with the application of CMC and metatartaric acid, with metatartaric acid leading to a greater increase in the stability of the treated wines (Table 2), consistent with other researchers [28,36,45]. However, dissimilar to cold stabilization, which significantly decreased tartaric acid concentration and potassium ions, the use of CMC did not reduce the levels of tartaric acid and potassium ions, as also noted by Bosso et al. [36]. No effect was observed on calcium, sodium, magnesium, or iron with any of the three treatments (Table 2). These results align with the extractive nature of cold stabilization, where lower temperatures induce the precipitation of potassium bitartrate when the concentration product exceeds the solubility of KHT [1]. Conversely, CMC and metatartaric acid act as colloidal protectors, inhibiting the precipitation of KHT by interfering with crystal growth [3,19].

### 3.2. Impact of Cold Stabilization, Carboxymethylcellulose, and Metatartaric Acid on the Wines Phenolic Composition

For white wine, none of the stabilization techniques affected the total phenolic compounds, total flavonoids, or total non-flavonoids (Table 3). The same was observed for the individual phenolic compounds determined by HPLC (Table 4). Additionally, the use of CMC and metatartaric acid did not result in a significant decrease or change in the phenolic composition of the wines, consistent with the findings of Guise et al. [17]. For rosé wine, cold stabilization resulted in a significant decrease in the total phenolic compounds and colored anthocyanins and a decrease, although not significant, in total anthocyanins and polymeric pigments (Table 3). HPLC analysis of the individual phenolic compounds and anthocyanins showed a significant decrease in the (+)-catechin content of the wines after cold stabilization (Table 4 and Table 5). A decrease was observed in C-3G, Pet-3G, M-3G, D-3AcGlc, M-3AcGlc, and M-3CGlc (Table 5).

The use of CMC and metatartaric acid for wine stabilization did not result in a significant change in the phenolic composition of rosé wine. A similar trend was observed for red wine, where cold stabilization resulted in a significant decrease in total phenolic compounds, total non-flavonoid phenolics, colored anthocyanins, polymeric pigments, and a decrease in total anthocyanins and total pigments. Analysis of the individual phenolic compounds, consistent with the observations for rosé wine, showed a significant decrease in (+)-catechin and C-3Glc, Pet-3Glc, Peo-3Glc, M-3Glc, Pet-3AcGlc, and Pet-3CGlc. These results are in line with Martínez-Pérez et al. [4], who also showed that red wine stabilized by cold treatment exhibited a decrease in color intensity. Again, the use of CMC and metatartaric acid did not result in any significant change in the phenolic composition of red wines (Table 3, Table 4 and Table 5), as also previously observed by Filipe-Ribeiro et al. [27].

### 3.3. Impact of Cold Stabilization, Carboxymethylcellulose, and Metatartaric Acid on the Wine Color and Chromatic Characteristics

In line with the variation in the phenolic composition of white wines with the different treatments applied, there was no significant change in the color of white wines measured at 420 nm. The same trend was observed for the chromatic parameters L* and b*, with a significant decrease in the a* value for wines treated by cold stabilization. However, the a* values were low for all white wines, resulting in similar C* values and no significant differences in the ∆E* value when compared with the use of CMC and metatartaric acid (Table 6). Similar results were obtained by Guise et al. [17]. For rosé wine, there was a significant decrease in color intensity, an increase in the L* value, and a decrease in the a* value, resulting in a significantly lower C* for wines treated by cold stabilization. Additionally, there was an increase in the h° and a significantly higher ∆E value compared with CMC and metatartaric acid. This decrease in color intensity, a* value, and C* value are attributable to a decrease in anthocyanins, colored anthocyanins, and polymeric pigments observed for rosé wines stabilized by cold treatment. For red wines, in line with the decrease in total anthocyanins, individual anthocyanins, colored anthocyanins, and polymeric pigments, a decrease in the color intensity of red wines treated with cold stabilization was observed. There was also an increase in the L* value and the a* value, with a significant decrease in the b* value, resulting in a significantly higher C*, a lower h°, and a significantly higher ∆E* value compared with the application of CMC and metatartaric acid (Table 6). Filipe-Ribeiro et al. [27] also did not observe significant differences in the L*, a*, b*, C*, and h° values in red wine after the addition of CMC and metatartaric acid.

### 3.4. Impact of Cold Stabilization, Carboxymethylcellulose, and Metatartaric Acid on the Wines Volatilome

#### 3.4.1. Wines Volatilome

The volatilomes of white, rosé, and red wines are presented in Table 7. A total of 32 compounds were detected in wines, some of which were common to all three types of wines (e.g., ethyl acetate, 3-methylbutanol acetate, 3-methylbutanol, ethyl hexanoate, hexyl acetate, ethyl octanoate, linalool, ethyl decanoate, 2-phenylethyl acetate, 2-phenylethanol, ethyl dodecanoate, octanoic acid, and decanoic acid), while other were unique to each wine. Twenty-three volatile organic compounds (VOCs) were identified either by comparison with standards or through comparison with the MS spectra obtained from the library and Kovats retention index (Table 7). The abundance of each volatile compound is expressed as the mean of the total ion current area. In white and rosé wines, esters were the most abundant compounds detected, and they were also present in high abundance in red wines. In fact, esters are considered the most frequently encountered VOCs in wine, though their abundance can vary. Esters are synthesized in grapes but rarely in significant amounts; the majority of wine esters are secondary or tertiary flavor compounds formed by the esterification of carboxylic acids (e.g., acetic acid) and alcohols (e.g., ethanol) [46]. The esters identified, analyzed, and quantified were as follows: ethyl acetate, 3-methyl-1-butanol acetate, ethyl hexanoate, hexyl acetate, ethyl octanoate, ethyl bezeneaceate, 2-phenylethanol acetate, ethyl decanoate, hexyl decanoate, ethyl dodecanoate, ethyl tetradecanoate, and ethyl hexadecanoate. Generally, the esters with higher concentrations in our wines were 3-methyl-1-butanol acetate, ethyl octanoate, and ethyl decanoate.

Several positive descriptors are associated with these compounds; for instance, 3-methyl-1-butanol acetate is linked to the positive attribute of “banana” [54], ethyl hexanoate to apple peel and fruity aromas [55], and ethyl octanoate to fruity, banana, or pineapple aromas [56]. Furthermore, ethyl dodecanoate, ethyl tetradecanoate, and ethyl hexadecanoate are known to impart sweet, waxy, and creamy aromas to wines [57]. Alcohols represent another important group of VOCs in the wines analyzed, being more abundant in red wine. Higher (fusel) alcohols are produced during alcoholic fermentation through the catabolism of amino acids and may influence the wine aroma both directly and indirectly [58,59,60]. A total of six alcohols were identified and quantified in this study: 3-methylbutanol, hexanol, benzyl alcohol, and 2-phenyl-ethanol (Table 7). These compounds are formed during alcoholic fermentation, and some are recognized by their strong and pungent smell and taste, contributing to herbaceous notes. Among individual compounds, 3-methyl-1-butanol, characterized by burnt or malty notes [61], had the highest concentration in all the studied wines. Another aroma with a significant impact on wines is 2-phenylethanol, synthesized via the Ehrlich pathway through metabolic reactions involving transamination of the amino acid L-phenylalanine, which can contribute a rosé note to the wine aroma [54]. In this group, the C6 alcohols (e.g., 1-hexanol), produced from the enzymatic oxidation of linolenic and linoleic acids in grape berries (lipoxygenase pathway), contribute to herbaceous and green aromas [59,62].

Acids were also detected in lower abundance. The acids identified in the wines analyzed were octanoic and decanoic acids. While these compounds are not primarily associated with wine quality, their presence plays an important role in the complexity of the aroma [63]. Many are thought to be responsible for “green” aromas in grape juice, although they may have less impact on wines [64]. Octanoic acid is associated with sweat and cheese aromas [65], and decanoic acid with fatty aromas [66]. Monoterpenes, sesquiterpenes, and C13-norisoprenoids, which derive from the grapes, are the main contributors to varietal character. Linalool and γ-terpinene found in the analyzed wines are responsible for varietal aromas [67] and may enhance the perception of fruity, citrus, and floral aromatic aromas in wines [68]. Linalool has aromas of lavender [65], while nerolidol imparts sweet and fruity aromas [47].

In red wines, volatile phenols such as 4-ethylphenol and 4-ethylguaiacol were also detected (Table 7). These volatile phenols negatively affect wine quality, being responsible for animal and smoky odors [69]. A known source of volatile phenols associated with off-odors is the spoilage yeast *Brettanomyces/Dekkera*, which produces 4-ethylphenol and 4-ethylguaiacol, imparting animal, horse stable, sweaty, and medicinal characteristics at excessive concentrations [70,71].

#### 3.4.2. Changes in White Wine Volatilome

A total of 21 VOCs were detected in the analyzed white wine, with 14 VOCs identified. Esters were the major group of compounds identified, accounting for 86% of the total volatilome, with ethyl decanoate being the most abundant ester. Alcohols (7.2%), including 3-methylbutanol and 2-phenylethanol; acids (5.6%), including octanoic and decanoic acids; and terpenols (1.2%), such as γ-terpinene and linalool, were also identified. Unknown compounds represented only 0.3% of the total volatilome. Changes in the abundance of VOCs were observed with the application of cold stabilization and the use of the enological stabilizers CMC and metatartaric acid (Table 7).

**Figure 1:**
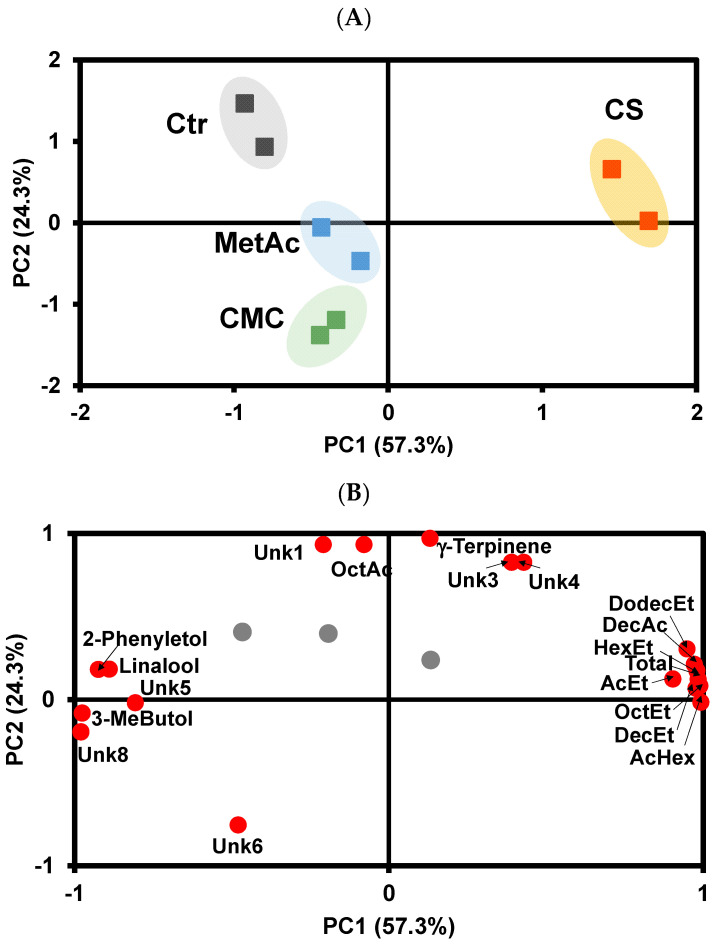
Sample scores projection on the first and second principal components (**A**) and variable loading on the first and second principal components of the effect of tartaric stabilization methods on the white wine volatilome (**B**). Ctr—Control wine, CS—Cold stabilization, CMC—Carboxymethylcellulose stabilization, MetAc—Metatartaric acid stabilization; AcEt—Ethyl acetate, AcHex—Hexyl acetate, OctEt—Ethyl octanoate, DecEt—Ethyl decanoate, HexEt—Ethyl hexanoate, DodecEt—Ethyl dodecanoate, OctAc—Octanoic acid; DecAc—Decanoic acid; 3-MeButol—3-Methyl butanol; 2-Phenyletol—2—Phenylethanol, Unk—Unknown. The gray circles are variables whose eigenvalues is less than 0.70.

To gain deeper insight into the internal structure of these data and the possible relationship between variables, a principal component analysis (PCA) was performed on these white wine volatilome data before and after tartaric stabilization treatment. PCA, after Varimax rotation of all VOCs, yielded three principal components explaining 92% of the total variance in the original data set. Loading values after Varimax rotation of >+0.70 and< −0.70 are marked in boldface type (PC1, PC2, and PC3) in Table S2 (Supplementary Materials). The first PC, which explains 57.3% of the total variance, correlates positively with Total VOCs, ethyl hexanoate, ethyldecanoate, ehtyloctanoate, ehtylacetate, hexylacetate, decanoic acid, and ethyldodecanoate, and negatively with 2-phenylethanol, linalool, 3-methylbutanol, and unknown VOCs 5 and 8 (Figure 1B). The second PC, which explains 24.3% of the total variance, correlates positively with γ-terpinene, octanoic acid, and unknown VOCs 1, 3, and 4 and negatively with unknown VOC 6.

The scatter plot of the sample scores on the first and second PC (Figure 1A) shows that white wines stabilized by cold stabilization presented positive values on PC1 and are clearly separated from control white wines and wines stabilized by the addition of the enological stabilizers CMC and metatartaric acid. Therefore, white wines stabilized by cold stabilization showed a higher abundance of total VOCs, related to an increase in the abundance of ethylhexanoate, ethyldecanoate, ehtyloctanoate, ehtylacetate, hexylacetate, decanoic acid, and ethyldodecanoate, compared with control wines and wines stabilized by the addition of CMC and metatartaric acid. On the other hand, wines stabilized by cold stabilization presented a lower abundance of 2-phenylethanol, linalool, 3-methylbutanol, and unknown VOCs 5 and 7 compared with the other wine samples.

Control wines and wines treated with CMC and metatartaric acid are differentiated according to PC2, with control wines presenting positive PC2 scores and white wines treated with CMC presenting negative PC2 scores. Research on the changes in volatile compounds in wines due to cold treatment is very limited. Xia et al. [72] studied the effect of cold treatment on Riesling dry white wines and found that it could promote the preservation of volatile compounds without negatively impacting the white wine aroma profile. White wines treated with metatartaric acid present PC2 scores near zero. Therefore, control white wines showed a higher abundance of octanoic acid, γ-terpinene, and unknown VOCs 1, 3, and 4 and a lower abundance of unknown VOC 6, with the reverse being true for white wines treated with CMC.

**Figure 2:**
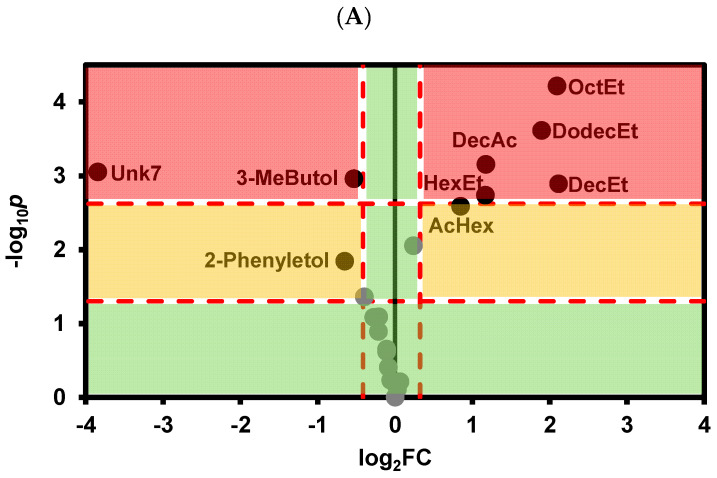

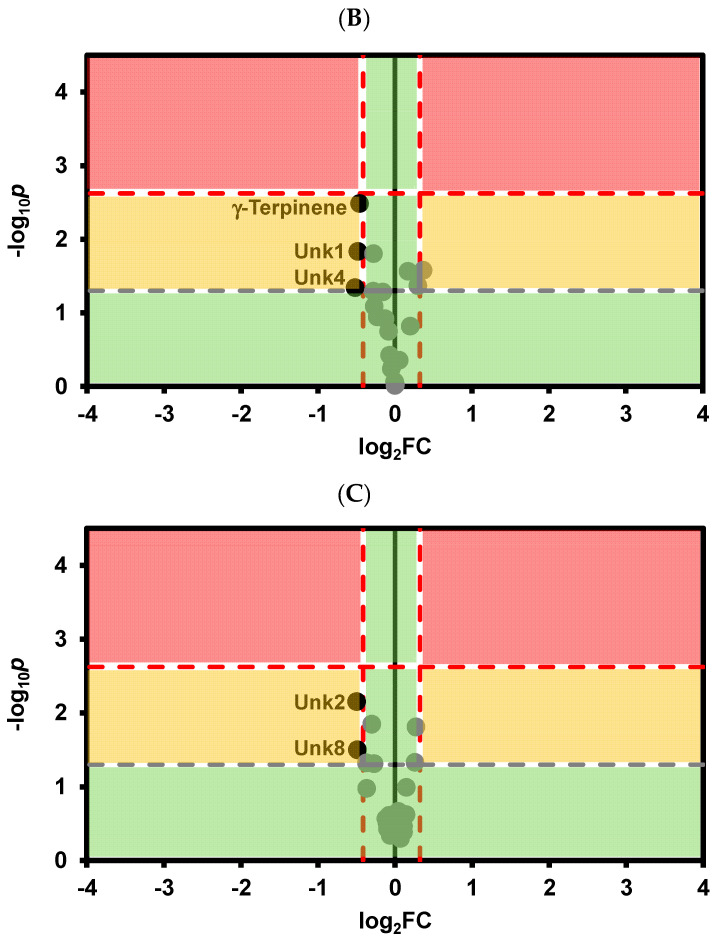
Volcano plot representing the statistical significance (*p*-values) from the *t*-Student test and the fold change (FC) for the relative VOC abundance of (**A**) Cold stabilization versus control wine; (**B**) CMC stabilization versus control wine; (**C**) Metatartaric acid stabilization versus control wines. The horizontal line represents the threshold of significance (*p* = 0.05) in gray color and after Bonferroni correction for multiple comparisons (*p* = 0.05/n) in red color. Vertical lines represent a 25% fold change in the VOC abundance. AcHex—Hexyl acetate, OctEt—Ethyl octanoate, DecEt—Ethyl decanoate, HexEt—Ethyl hexanoate, DodecEt—Ethyl dodecanoate, DecAc—Decanoic acid; 3-MeButol—3-Methyl butanol; 2-Phenyletol—2—Phenylethanol, Unk—Unknown. The green circles correspond to variables with non significant differences in relation to control wine and, or with less than 25% fold change in relation to control wine.

To better visualize the differences in the abundance of individual VOCs between control white wines and wines after tartaric stabilization by cold stabilization treatment, as well as the addition of CMC and metatartaric acid, and to assess the magnitude of these differences, Volcano plots were used (Figure 2A–C). As shown in Figure 2, cold stabilization resulted in a greater difference in VOC abundance compared with treatments using the enological stabilizers CMC and metatartaric acid. In accordance with the PCA results, there was a significant increase in the abundance of ethyl esters of octanoic, decanoic, and dodecanoic acids, ethylhexanoate, hexylacetate, and decanoic acid. Conversely, there was a significant decrease in 3-methylbutanol, 2-phenylbutanol, and the unknown VOC 8. For CMC, only a significant decrease in γ-terpinene and unknown VOC 1 and 4 was observed, while for metatartaric acid, only a decrease in unknown VOCs 2 and 8 was observed compared with control wine.

#### 3.4.3. Changes in Rosé Wine Volatilome

**Figure 3:**
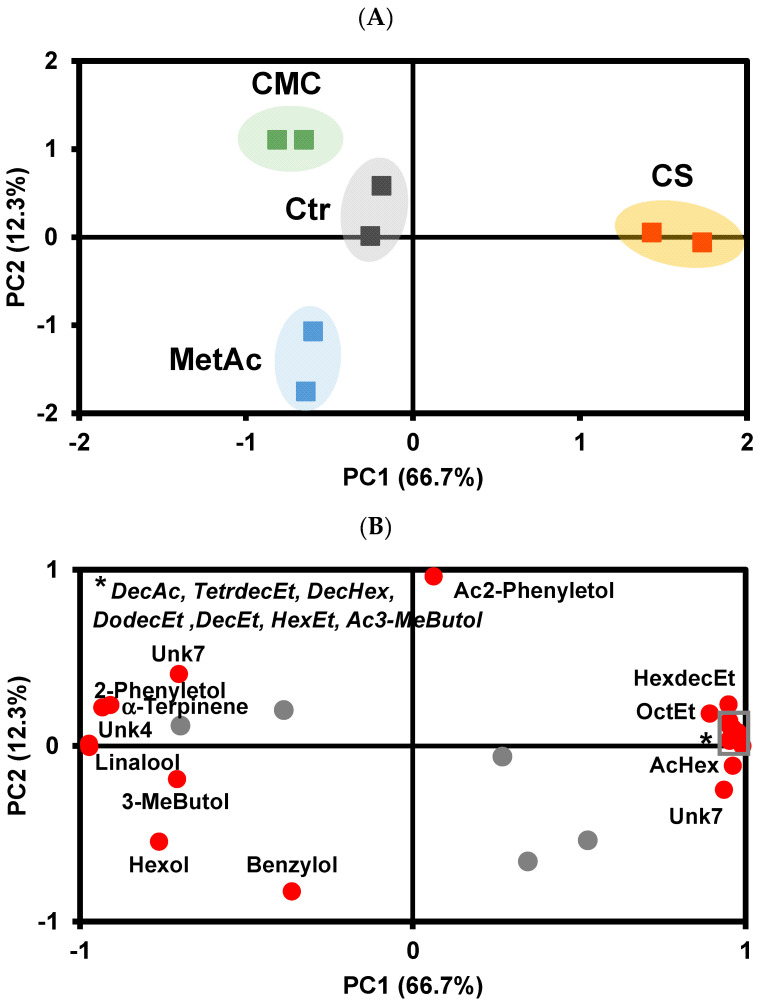
Sample scores projection on the first and second principal components (**A**) and variable loading on the first and second principal components of the effect of tartaric stabilization methods on the rosé wine volatilome (**B**). Ctr—Control wine, CS—cold stabilization, CMC—carboxymethylcellulose stabilization, MetAc—metatartaric acid stabilization; AcHex—Hexyl acetate, OctEt—ethyl octanoate, DecEt—ethyl decanoate, HexEt—ethyl hexanoate, DodecEt—Ethyl dodecanoate, DecAc—decanoic acid; 3-MeButol—3-Methyl butanol; 2-Phenyletol—2—Phenylethanol, TetrdecEt-Ethyl tetradeconoate, DecHex—Hexyl decanoate, Ac3-MeButol-3-Methylbutanol acetate, Ac2-Phenyletol-2-Phenylethyl acetate, Hexol—Hexanol, Benzylol—Benzyl alcohol, HexdecEt-Ethyl hexadecanoate Unk—Unknown. The gray circles are variables whose eigenvalues is less than 0.70. The gray square outlines the compounds marked with ‘*’.

For rosé wine, a total of 25 VOCs were detected, with 19 of them identified. As with white wine, esters were the most abundant VOCs, representing 95% of the total volatilome, followed by alcohols (3.2%), acids (1.2%), and terpenes (0.2%), with unknowns accounting for only 0.03% of the total volatilome. In rosé wine, ethyl octanoate was the most abundant ester, while 3-methylbutanol and 2-phenylethanol were the most abundant alcohols, and octanoic acid was the predominant acid. Similar to what was observed in white wine, the cold stabilization of rosé wines resulted in a significant change in the volatilome (Table 7). PCA analysis after Varimax rotation (Tables S4–S6, Supplementary Materials) shows that, such as in white wine, rosé wines stabilized by cold stabilization presented a higher abundance of total VOCs compared with control rosé wine and wines stabilized by the addition of CMC and metatartaric acid. This increase in total volatilome is attributable to a rise in esters, except for acetyl 2-phenylethanol, compared with the other wines, and lower amounts of alcohols, terpenes, and unknown VOC 7. Wines treated with CMC are distinguished from those treated with metatartric acid according to PC2 (Figure 3A), showing a higher abundance of benzyl alcohol and a lower abundance of acetyl 2-phenylethanol compared with the wines treated with metatartaric acid.

**Figure 4:**
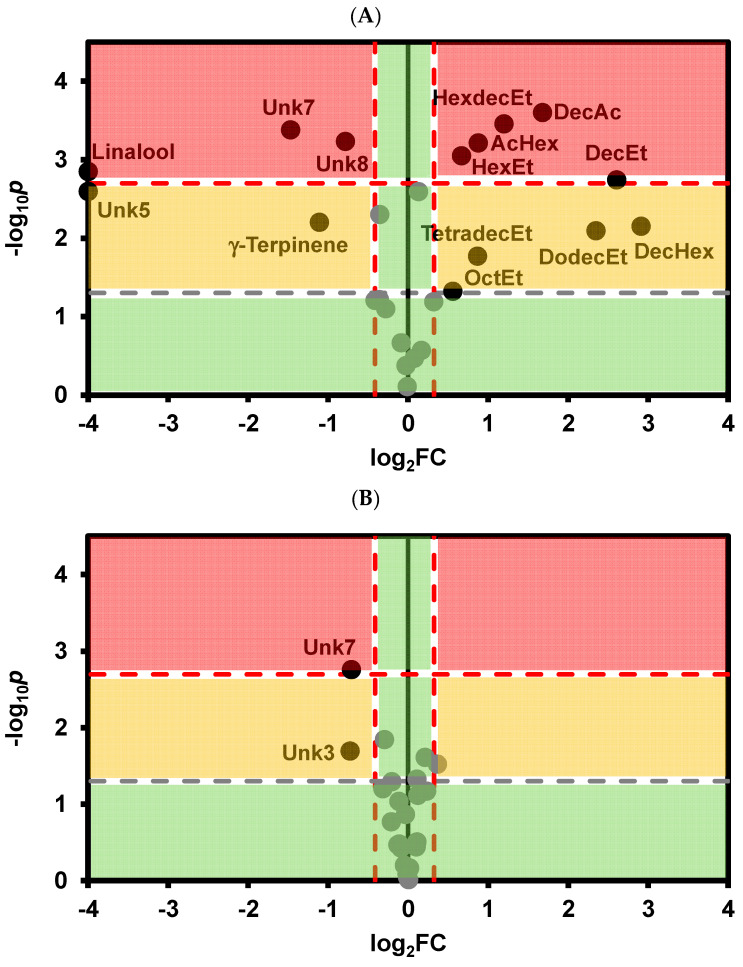

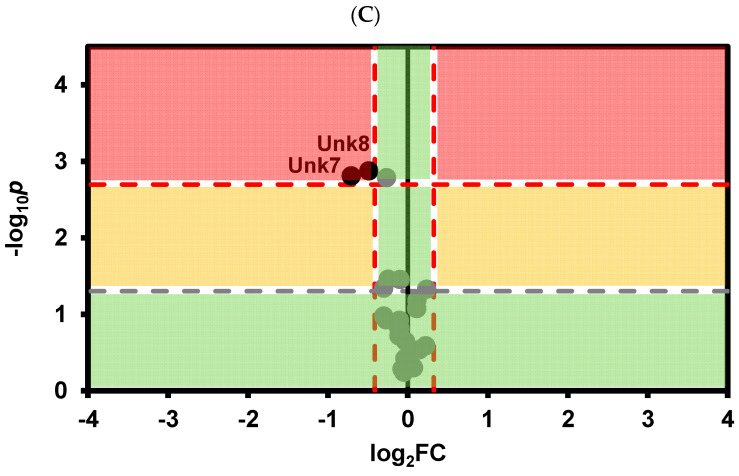
Volcano plot representing the statistical significance (*p*-values) on the *t*-Student test and the fold change (FC) for the relative VOC abundance of (**A**) Cold stabilization versus control rosé wine; (**B**) CMC stabilization versus control rosé wine; (**C**) Metatartaric acid stabilization versus control rosé wines. The horizontal line represents the threshold of significance (*p* = 0.05) in gray color and after Bonferroni correction for multiple comparisons (*p* = 0.05/n) in red color. Vertical lines represent a 25% fold change in the VOC abundance. AcHex—Hexyl acetate, OctEt—Ethyl octanoate, DecEt—ethyl decanoate, HexEt—ethyl hexanoate, DodecEt—Ethyl dodecanoate, DecAc—decanoic acid; TetrdecEt-Ethyl tetradeconoate, HexdecEt-Ethyl hexadecanoate, Unk—Unknown. The green circles correspond to variables with non significant differences in relation to control wine and, or with less than 25% fold change in relation to control wine.

Rosé wine treated with cold stabilization showed significantly higher amounts of ethyl decanoate, decanoic acid, hexylacetate, ethyl hexadecanoate, ethyl hexanoate, ethyl octanoate, ethyl dodecanoate, ethyl tetradecanoate, and hexyl decanoate, along with lower levels of γ-terpinene, linalool, and unknown VOCs 5, 7 and 8 (Figure 4A). For CMC and metatartaric acid, significant differences were observed only in unknown VOCs 3 and 7 and unknown VOCs 7 and 8, respectively, compared with the control wine (Figure 4B,C).

#### 3.4.4. Changes in Red Wine Volatilome

The volatilome of red wines differed notably from that of white and rosé wine. First, the total VOC abundance in red wine was significantly lower compared with white and rosé wines. In red wine, 22 VOCs were detected, and 21 were identified (Table 7). Dissimilar to white and rosé wines, alcohols were the most abundant VOCs in red wine (54%), with 2-phenylthexanol being the most prevalent among them. Esters accounted for 43% of the total VOCs, with ethyl octanoate being the most abundant ester. Additionally, acids represented 1.7%, terpene (the only one detected), such as linalool, accounted for 1.3%, and unknown VOCs represented 0.02%. Volatile phenols, including 4-ethylphenol, 4-ethylguaiacol, and 2-nonanone, together made up 0.09% of the total VOCs detected (Table 7).

**Figure 5:**
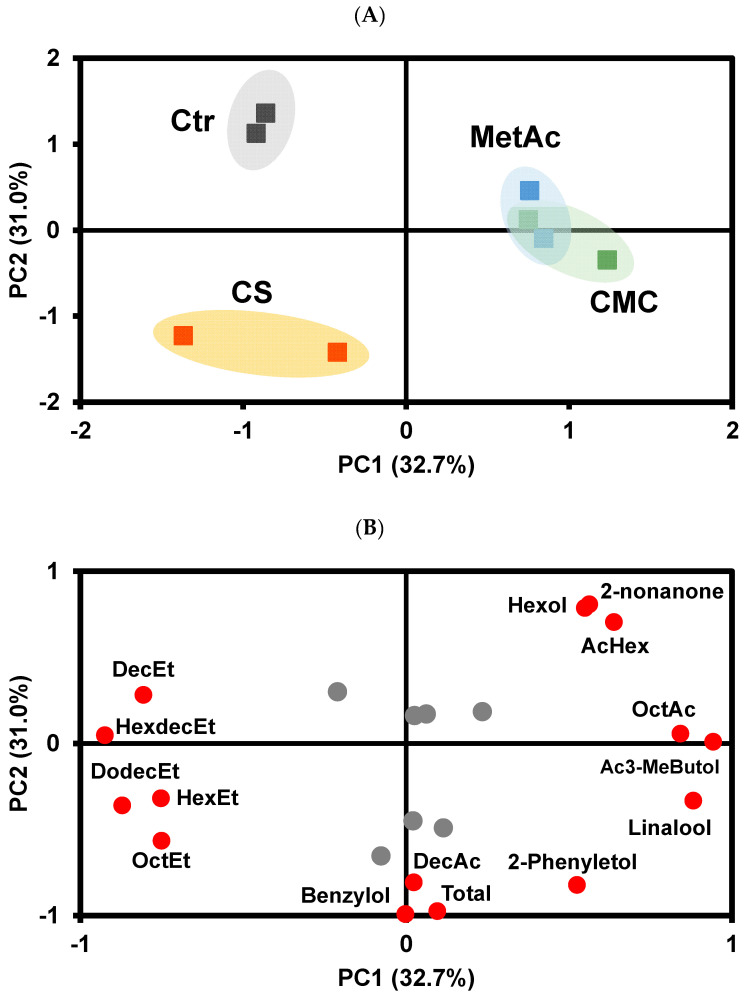
Sample scores projection on the first and second principal components (**A**) and variable loading on the first and second principal components of the effect of tartaric stabilization methods on the red wine volatilome (**B**). Ctr—Control wine, CS—cold stabilization, CMC—carboxymethylcellulose stabilization, MetAc—metatartaric acid stabilization; OctEt—ethyl octanoate, DecEt—ethyl decanoate, HexEt—ethyl hexanoate, DodecEt—Ethyl dodecanoate, DecAc—decanoic acid; 2-Phenyletol—2—phenylethanol, HexdecEt-Ethyl hexadecanoate, Benzylol—Benzyl alcohol, Ac3-MeButol-3-Methylbutanol acetate, Unk—Unknown. The gray circles are variables whose eigenvalues is less than 0.70.

**Figure 6:**
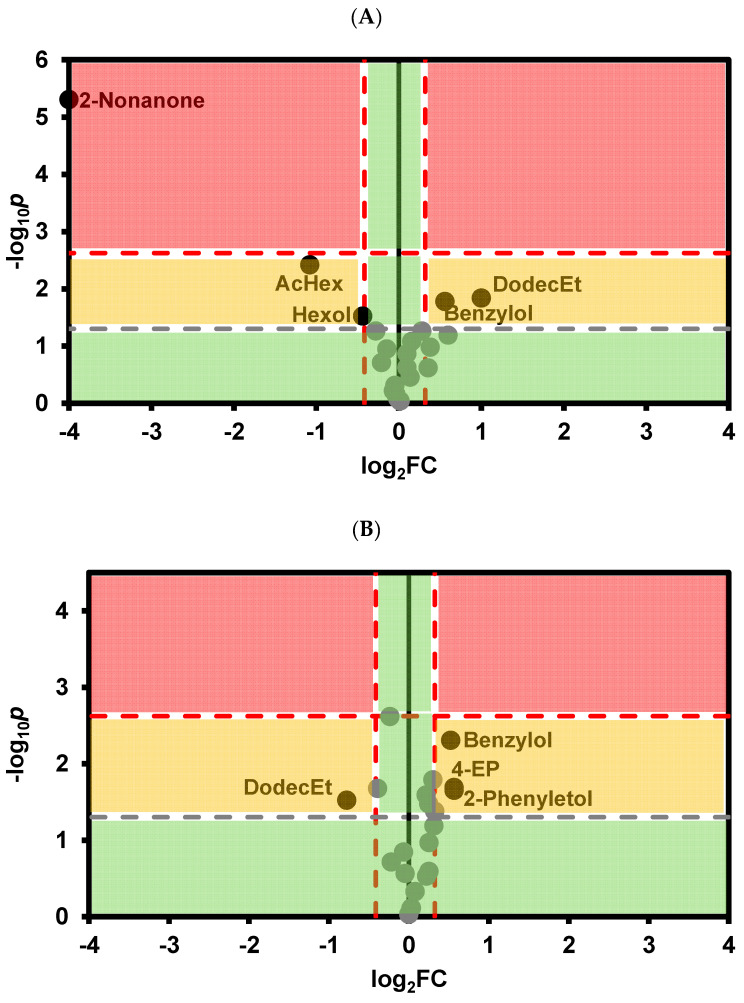

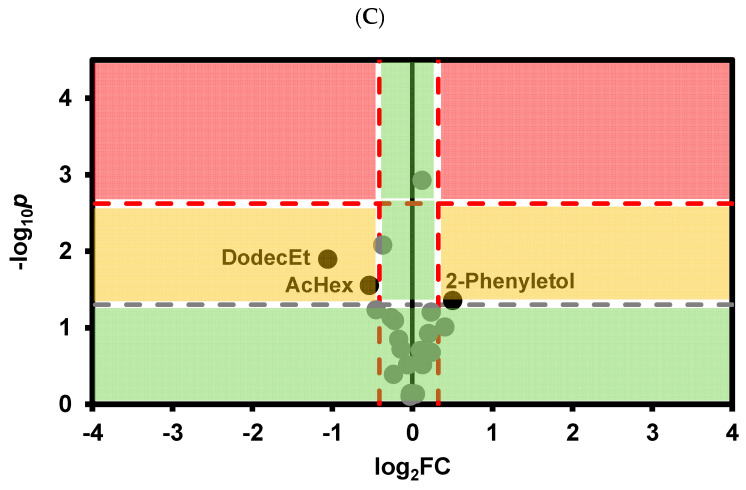
Volcano plot representing the statistical significance (*p*-values) on the *t*-Student test and the fold change (FC) for the relative VOC abundance of (**A**) Cold stabilization versus control red wine; (**B**) CMC stabilization versus control red wine; (**C**) Metatartaric acid stabilization versus control red wines. The horizontal line represents the threshold of significance (*p* = 0.05) in gray color and after Bonferroni correction for multiple comparisons (*p* = 0.05/n) in red color. Vertical lines represent a 25% fold change in the VOC abundance. DodecEt—Ethyl dodecanoate, 2-Phenyletol—2—phenylethanol, Benzylol—Benzyl alcohol, Hexol—Hexanol, AcHex—Hexyl acetate, 4-EP-4-Ethylphenol Unk—Unknown. The green circles correspond to variables with non significant differences in relation to control wine and, or with less than 25% fold change in relation to control wine.

As with white and rosé wine, cold stabilization treatment in red wines led to an increase in total VOC abundance (Table 7) and resulted in a distinct VOC profile compared with control wines and wines treated with CMC and metatartaric acid (Figure 5A, Tables S7–S9, Supplementary Materials). This difference was mainly due to a higher abundance of decanoic acid, benzyl alcohol, and 2-phenylethanol and a lower abundance of hexanol, 2-nonanone, and hexylacetate, compared with control wine, with samples being separated according to PC2 (Figure 5A,B). This observation is supported by the Volcano plot (Figure 6A).

Interestingly, red wines treated with CMC or metatartaric acid were not separated in the PCA analysis (Figure 5A) but were clearly distinct from control red wine based on the PC1 value (Figure 5A). CMC treated wines had significantly higher levels of benzyl alcohol, 2-phenylethanol, and 4-ethyl phenol and lower levels of ethyl dodecanoate compared with control wine (Figure 6B). In contrast, wines treated with metatartaric acid exhibited significantly higher levels of 2-phenylethanol and lower levels of ethyl dodecanoate and hexyl acetate compared with the control wines (Figure 6C).

The differences observed in the impact of cold stabilization among the three types of wines can be attributed to the lower abundance of esters in red wine compared with white and rosé wines. Esters are the compounds most affected in terms of abundance when wines undergo cold stabilization. The distribution and proportion of aroma compounds in wine and its headspace primarily depend on their volatility, which is influenced by the matrix composition, including polyphenols, ethanol, polysaccharides, proteins, and the presence of aroma compounds [73,74,75]. The interaction between aroma compounds and polyphenols has received increasing attention because of its impact on odorant volatility and aroma release [76]. The structural properties and concentration of both aroma and phenolic compounds significantly affect the behavior of wine aroma release [76].

The results indicate that regardless of the wine matrix—whether white, rosé, or red—cold stabilization significantly alters the volatilome of wines, much more so than the application of enological stabilizers for tartaric stabilization, which only slightly impacts the volatilome. The observed increase in ester VOCs in white and rosé wines treated by cold stabilization may be due to a decrease in total alcohols [77]. For these wines, there is a significant decrease in total alcohols and a significant increase in total esters (Table 7). In contrast, the smaller differences observed in red wines regarding total esters can be explained by the smaller changes in total alcohols, which are present in higher abundance compared with white and rosé wines (Table 7).

As discussed earlier, the cold treatment of rosé and red wines decreased the concentration of total phenols, including anthocyanins and catechins. It has also been shown that increasing catechin concentrations reduces the volatility of compounds such as isoamyl acetate, ethyl hexanoate, and benzaldehyde [78]. The impact on the sensory profile of wines is not straightforward; an increase in esters abundance in the headspace would typically enhance the fruity aroma, which is generally positive. However, since these compounds are less integrated with the wine matrix, they may lose their aroma more rapidly over time. This is an important aspect that should be explored further in future studies.

## 4. Conclusions

To our knowledge, this is the first study to evaluate and compare the impact of cold stabilization and common enological additives used for wine tartrate stabilization on the volatilome of white, rosé, and red wines. The use of enological stabilizers had minimal to no impact on the volatilome of these wines compared with cold stabilization, indicating that the expected influence of stabilizers on wine aroma characteristics is negligible or nonexistent. The sensory impact observed on the volatilome of cold-stabilized wines is complex. For white and rosé wines, an increase in sensory-positive ester VOCs was noted, along with a decrease in higher alcohols, which can enhance the wine aroma. This aspect requires further research. In red wines, the impact on the volatilome was more limited, as these wines typically contain a lower abundance of esters. Additionally, cold stabilization affects the phenolic composition of rosé and red wines, resulting in a decrease in red color. Therefore, while cold stabilization has a more significant effect on the sensory characteristics of wines, this effect is mixed: it negatively impacts the color of rosé and red wines but potentially enhances the aroma.

## Figures and Tables

**Table 1:** Physicochemical characteristics of the white, rosé, and red wines used in the study.

Parameters	White	Rosé	Red
Alcohol strengths (% *v*/*v*)	12.7	12.5	11.6
Density at 20 °C (g/mL)	0.9885	0.9988	0.9949
Titratable acidity (g/L tartaric acid)	6.7	5.8	6.5
pH	3.08	3.52	3.61
Volatile acidity (g/L acetic acid)	0.20	0.22	0.45
Total sulphur dioxide (mg/L)	96	106	59
Free sulfur dioxide (mg/L)	27	23	28
Malic acid (g/L)	1.59	2.57	-
Reducing sugars (g/L)	2.4	3.3	4.2
Tartaric instability Δx (µS/cm)	159.3	100.8	70.5

**Table 2:** Concentration of tartaric acid, tartaric stability, and minerals in white, rosé, and red wine after different tartaric stabilization treatments (mean (mean ± standard deviation).

	Tartaric Acid (g/L)	Tartaric Stability Δx (μS/cm)	Potassium (mg/L)	Calcium (mg/L)	Sodium (mg/L)	Magnesium (mg/L)	Iron (mg/L)
White wine							
Ctr	3.624 ± 0.003 ^a^	159.3 ± 9.7 ^a^	763.4 ± 5.7 ^a^	51.9 ± 0.3 ^a^	73.7 ± 0.3 ^a^	62.5 ± 0.9 ^a^	0.85 ± 0.07 ^a^
CS	2.964 ± 0.030 ^b^	31.5 ± 2.1 ^b^	551.3 ± 17.1 ^b^	51.4 ± 1.0 ^a^	72.2 ± 0.2 ^a^	65.9 ± 2.4 ^a^	0.80 ± 0.14 ^a^
CMC	3.603 ± 0.030 ^a^	46.5 ± 3.0 ^c^	725.1 ± 65.7 ^a^	49.4 ± 1.7 ^a^	84.0 ± 2.7 ^b^	58.4 ± 2.6 ^a^	0.80 ± 0.00 ^a^
MetAc	3.539 ± 0.000 ^a^	11.5 ± 0.7 ^d^	680.5 ± 2.7 ^a^	52.1 ± 0.7 ^a^	73.0 ± 1.0 ^a^	61.0 ± 0.1 ^a^	0.95 ± 0.07 ^a^
Rosé wine							
Ctr	2.602 ± 0.000 ^a^	100.8 ± 3.9 ^a^	1031.1 ± 18.2 ^a^	43.2 ± 2.6 ^a^	77.6 ± 0.2 ^a^	68.5 ± 7.2 ^a^	1.20 ± 0.07 ^a^
CS	2.347 ± 0.060 ^b^	48.8 ± 3.4 ^b^	934.4 ± 22.6 ^a^	45.7 ± 2.6 ^a^	74.6 ± 0.4 ^a^	70.4 ± 4.5 ^a^	1.00 ± 0.07 ^a^
CMC	2.581 ± 0.030 ^a^	23.0 ± 2.2 ^c^	1016.9 ± 34.8 ^a^	43.7 ± 1.6 ^a^	81.6 ± 3.7 ^b^	71.6 ± 4.2 ^a^	1.10 ± 0.04 ^a^
MetAc	2.602 ± 0.060 ^a^	14.0 ± 0.0 ^d^	943.4 ± 7.3 ^a^	42.7 ± 0.7 ^a^	72.1 ± 1.8 ^a^	69.9 ± 1.1 ^a^	1.30 ± 0.14 ^a^
Red wine							
Ctr	3.262 ± 0.030 ^a^	70.5 ± 0.7 ^a^	1204.1 ± 30.6 ^a^	71.8 ± 4.9 ^a^	58.1 ± 0.1 ^a^	118.2 ± 13.4 ^a^	0.95 ± 0.07 ^a^
CS	2.986 ± 0.060 ^a^	31.5 ± 3.1 ^b^	1328.6 ± 68.1 ^b^	68.4 ± 4.2 ^a^	61.2 ± 0.7 ^a^	110.1 ± 6.6 ^a^	0.80 ± 0.00 ^a^
CMC	3.390 ± 0.090 ^a^	38.0 ± 2.2 ^c^	1385.0 ± 3.9 ^b,c^	67.9 ± 1.4 ^a^	70.1 ± 4.7 ^b^	112.5 ± 5.8 ^a^	0.75 ± 0.07 ^a^
MetAc	3.390 ± 0.090 ^a^	19.5 ± 1.3 ^d^	1431.8 ± 11.8 ^b,d^	68.9 ± 0.7 ^a^	58.2 ± 2.6 ^a^	119.8 ± 2.0 ^a^	0.95 ± 0.21 ^a^

Control wine (Ctr), Wine treated by cold stabilization (CS), Wine treated with carboxymethylcellulose (CMC), and Wine treated with metatartaric acid (MetA). Columns for each wine followed by the same letter do not present statistically significant differences (Tukey, 5%).

**Table 3:** Phenolic composition of white, rosé, and red wine, and the browning potential in white wine after different tartaric stabilization treatments (mean ± standard deviation).

	Total Phenolics (mg/L GAE)	Total Flavonoids (mg/L GAE)	Total Non-Flavonoids (mg/L GAE)	Total Anthocyanins (mg/L)	Colored Anthocyanins (a.u.)	Polymeric Pigments (a.u.)	Total Pigments (a.u.)	Browning Potential (a.u.)
White wine								
Ctr	178.8 ± 0.0 ^a^	57.9 ± 3.3 ^a^	121.0 ± 3.3 ^a^	-	-	-	-	0.0020 ± 0.0014 ^a^
CS	177.3 ± 2.2 ^a^	57.3 ± 2.4 ^a^	120.0 ± 0.3 ^a^	-	-	-	-	0.0025 ± 0.0021 ^a^
CMC	178.8 ± 4.3 ^a^	59.0 ± 3.3 ^a^	119.8 ± 7.6 ^a^	-	-	-	-	0.0015 ± 0.0007 ^a^
MetAc	178.8 ± 0.0 ^a^	59.0 ± 0.0 ^a^	119.8 ± 0.0 ^a^	-	-	-	-	0.0020 ± 0.0014 ^a^
Rosé wine								
Ctr	237.1 ± 4.3 ^a^	58.4 ± 0.8 ^a^	178.7 ± 5.2 ^a^	11.8 ± 0.6 ^a^	0.067 ± 0.001 ^a^	0.050 ± 0.000 ^a^	0.56 ± 0.07 ^a^	-
CS	204.9 ± 2.2 ^b^	59.0 ± 0.0 ^a^	145.9 ± 2.2 ^b^	9.2 ± 0.6 ^a^	0.038 ± 0.007 ^b^	0.035 ± 0.007 ^a^	0.61 ± 0.00 ^a^	-
CMC	235.6 ± 2.2 ^a^	60.2 ± 1.6 ^a^	175.4 ± 0.5 ^a^	10.9 ± 0.6 ^a^	0.061 ± 0.006 ^a^	0.055 ± 0.007 ^a^	0.56 ± 0.07 ^a^	-
MetAc	238.7 ± 2.2 ^a^	60.2 ± 0.0 ^a^	178.5 ± 2.2 ^a^	11.4 ± 0.0 ^a^	0.066 ± 0.002 ^a^	0.050 ± 0.000 ^a^	0.61 ± 0.00 ^a^	-
Red wine								
Ctr	530.1 ± 2.2 ^a^	73.4 ± 0.8 ^a^	456.7 ± 1.4 ^a^	154.4 ± 3.1 ^a^	1.840 ± 0.014 ^a^	3.580 ± 0.014 ^a^	15.20 ± 1.21 ^a^	-
CS	497.9 ± 0.0 ^b^	72.8 ± 1.6 ^a^	425.0 ± 1.6 ^b^	149.6 ± 1.2 ^a^	1.670 ± 0.028 ^b^	3.325 ± 0.007 ^b^	13.84 ± 0.00 ^a^	-
CMC	528.5 ± 0.0 ^a^	72.8 ± 1.6 ^a^	455.7 ± 1.6 ^a^	152.7 ± 1.9 ^a^	1.86 ± 0.042 ^a^	3.570 ± 0.000 ^a^	14.40 ± 0.50 ^a^	-
MetAc	531.6 ± 4.3 ^a^	72.8 ± 0.0 ^a^	458.8 ± 4.3 ^a^	152.7 ± 1.9 ^a^	1.845 ± 0.035 ^a^	3.585 ± 0.007 ^a^	15.71 ± 0.21 ^a^	-

Control wine (Ctr), Wine treated by cold stabilization (CS), Wine treated with carboxymethylcellulose (CMC), and Wine treated with metatartaric acid (MetA). Columns for each wine followed by the same letter do not present statistically significant differences (Tukey, 5%), a.u.—absorbance units.

**Table 4:** Concentration of (+)-catechin and phenolic acids (mg/L) in white, rosé, and red wine after different tartaric stabilization treatments (mean ± standard deviation).

	Gallic Acid	(+)-Catechin	*trans*-Caftaric Acid	GRP	Coutaric Acid	Caffeic Acid	*p*-Coumaric Acid	Ferulic Acid	Ethyl Ester of Caffeic Acid	Ethyl Ester of Coumaric Acid
White wine										
Ctr	0.19 ± 0.00 ^a^	6.08 ± 0.47 ^a^	4.55 ± 0.28 a	0.09 ± 0.00 ^a^	0.48 ± 0.03 ^a^	1.14 ± 0.06 ^a^	0.35 ± 0.05 ^a^	0.41 ± 0.02 a	0.51 ± 0.00 ^a^	0.10 ± 0.01 ^a^
CS	0.19 ± 0.00 ^a^	6.60 ± 0.00 ^a^	4.49 ± 0.01 ^a^	0.06 ± 0.01 ^a^	0.49 ± 0.04 ^a^	1.15 ± 0.05 ^a^	0.40 ± 0.03 ^a^	0.42 ± 0.04 a	0.56 ± 0.05 ^a^	0.12 ± 0.03 ^a^
CMC	0.20 ± 0.02 ^a^	6.04 ± 0.51 ^a^	4.73 ± 0.03 ^a^	0.06 ± 0.01 ^a^	0.55 ± 0.01 ^a^	1.23 ± 0.04 ^a^	0.3 8 ± 0.02 ^a^	0.44 ± 0.05 a	0.53 ± 0.02 ^a^	0.11 ± 0.01 ^a^
MetAc	0.20 ± 0.00 ^a^	6.52 ± 0.01 ^a^	5.01 ± 0.39 ^a^	0.07 ± 0.02 a	0.59 ± 0.05 ^a^	1.19 ± 0.05 ^a^	0.36 ± 0.01 ^a^	0.43 ± 0.02 a	0.53 ± 0.02 ^a^	0.11 ± 0.00 ^a^
Rosé wine										
Ctr	0.30 ± 0.01 ^a^	8.78 ± 0.04 ^a^	2.32 ± 0.19 ^a^	0.16 ± 0.04 ^a^	0.58 ± 0.05 ^a^	1.56 ± 0.08 ^a^	0.48 ± 0.11 ^a^	0.46 ± 0.01 a	0.53 ± 0.01 ^a^	0.20 ± 0.01 ^a^
CS	0.28 ± 0.00 ^a^	5.62 ± 0.12 ^b^	2.05 ± 0.30 ^a^	0.16 ± 0.00 ^a^	0.65 ± 0.04 ^a^	1.61 ± 0.00 ^a^	0.50 ± 0.14 ^a^	0.47 ± 0.03 a	0.42 ± 0.07 ^a^	0.21 ± 0.04 ^a^
CMC	0.29 ± 0.00 ^a^	8.73 ± 0.31 ^a^	2.66 ± 0.03 ^a^	0.15 ± 0.02 ^a^	0.63 ± 0.06 ^a^	1.47 ± 0.03 ^a^	0.51 ± 0.05 ^a^	0.45 ± 0.02 a	0.44 ± 0.06 ^a^	0.21 ± 0.06 ^a^
MetAc	0.29 ± 0.01 ^a^	8.74 ± 0.08 ^a^	2.29 ± 0.24 ^a^	0.16 ± 0.01 ^a^	0.58 ± 0.15 ^a^	1.57 ± 0.07 ^a^	0.50 ± 0.06 ^a^	0.44 ± 0.01 a	0.47 ± 0.08 ^a^	0.19 ± 0.02 ^a^
Red wine										
Ctr	4.59 ± 0.81 ^a^	19.54 ± 3.23 ^a^	17.30 ± 2.22 ^a^	0.73 ± 0.34 ^a^	4.73 ± 0.98 ^a^	-	0.73 ± 0.12 ^a^	3.99 ± 0.25 a	2.04 ± 0.12 ^a^	0.32 ± 0.21 ^a^
CS	4.58 ± 0.48 ^a^	13.03 ± 2.00 ^b^	19.20 ± 0.12 ^a^	0.41 ± 0.27 ^a^	4.91 ± 0.58 ^a^	-	0.90 ± 0.04 ^a^	4.21 ± 0.60 a	1.91 ± 0.37 ^a^	0.24 ± 0.06 ^a^
CMC	5.95 ± 1.12 ^a^	23.78 ± 2.19 ^a^	19.41 ± 2.25 ^a^	0.27 ± 0.05 ^a^	5.94 ± 0.23 ^a^	-	0.85 ± 0.27 ^a^	4.32 ± 0.09 a	1.63 ± 0.05 ^a^	0.36 ± 0.14 ^a^
MetAc	5.55 ± 0.39 ^a^	20.40 ± 1.75 ^a^	17.54 ± 3.53 ^a^	0.67 ± 0.48 ^a^	5.42 ± 0.85 ^a^	-	0.84 ± 0.05 ^a^	4.31 ± 0.54 a	1.73 ± 0.32 ^a^	0.33 ± 0.15 ^a^

Control wine (Ctr), Wine treated by cold stabilization (CS), Wine treated with carboxymethylcellulose (CMC), Wine treated with metatartaric acid (MetA), GRP—2-*S*-glutathionylcaftaric acid. Columns for each wine followed by the same letter do not present statistically significant differences (Tukey, 5%).

**Table 5:** Concentration of monomeric anthocyanins (mg/L) in rosé and red wine after different tartaric stabilization treatments (mean ± standard deviation).

	D-3-Glc	C-3-Glc	Pet-3-Glc	Peo-3-Glc	M-3-Glc	D-3-AGlc	Pet-3-AGlc	Peo-3-AGlc	M-3-AGlc	C-3-CGlc	Pet-3-CGlc	Mal-3-CGlc
Rosé wine												
Ctr	0.027 ± 0.001 ^a,b,c^	0.145 ± 0.011 ^a^	0.263 ± 0.012 ^a^	0.198 ± 0.022 ^a^	1.668 ± 0.134 _a_	0.019 ± 0.001 ^a,b,c^	0.055 ± 0.008 ^a^	0.073 ± 0.009 ^a^	0.166 ± 0.010 ^a^			0.075 ± 0.001 ^a^
CS	0.022 ± 0.001 ^b^	0.087 ± 0.007 ^b^	0.185 ± 0.013 ^b^	0.162 ± 0.027 ^a^	1.211 ± 0.013 ^b^	0.016 ± 0.001 ^b^	0.046 ± 0.001 ^a^	0.044 ± 0.019 ^a^	0.127 ± 0.009 ^b^			0.057 ± 0.001 ^b^
CMC	0.029 ± 0.001 ^c^	0.134 ± 0.014 ^a^	0.303 ± 0.019 ^a^	0.190 ± 0.009 ^a^	1.521 ± 0.035 ^a^	0.018 ± 0.000 ^a,b,c^	0.057 ± 0.007 ^a^	0.061 ± 0.003 ^a^	0.164 ± 0.005 ^a^			0.070 ± 0.007 ^a^
MetAc	0.027 ± 0.002 ^a,b,c^	0.141 ± 0.005 ^a^	0.278 ± 0.013 ^a^	0.182 ± 0.000 ^a^	1.567 ± 0.027 ^a^	0.021 ± 0.001 ^c^	0.056 ± 0.000 ^a^	0.072 ± 0.003 ^a^	0.156 ± 0.005 ^a^			0.073 ± 0.002 ^a^
Red wine												
Ctr	2.16 ± 0.26 ^a^	50.86 ± 3.15 ^a^	50.72 ± 2.00 ^a^	36.03 ± 1.54 ^a^	154.16 ± 8.18 ^a^	5.49 ± 0.01 ^a^	1.65 ± 0.05 ^a^	5.10 ± 0.16 ^a,b,c^	11.70 ± 0.46 ^a^	0.71 ± 0.25 ^a^	2.43 ± 0.07 ^a^	12.35 ± 0.48 ^a^
CS	1.62 ± 0.21 ^a^	37.21 ± 0.86 ^b^	33.98 ± 1.89 ^b^	25.17 ± 0.36 ^b^	114.58 ± 4.26 ^b^	4.42 ± 0.43 ^a^	0.94 ± 0.15 ^b^	4.29 ± 0.11 ^b^	9.34 ± 0.83 ^a^	0.67 ± 0.05 ^a^	1.92 ± 0.12 ^b^	10.87 ± 1.16 ^a^
CMC	2.42 ± 0.67 ^a^	51.69 ± 3.14 ^a^	49.70 ± 3.43 ^a^	33.51 ± 3.47 ^a^	151.53 ± 5.86 ^a^	5.61 ± 0.47 ^a^	1.73 ± 0.06 ^a^	5.43 ± 0.34 ^c^	11.29 ± 1.31 ^a^	0.80 ± 0.25 ^a^	2.59 ± 0.17 ^a^	12.32 ± 0.25 ^a^
MetAc	2.16 ± 0.11 ^a^	48.89 ± 0.21 ^a^	47.94 ± 1.89 ^a^	34.59 ± 1.42 ^a^	147.36 ± 3.01 ^a^	5.30 ± 0.38 ^a^	1.67 ± 0.26 ^a^	5.09 ± 0.29 ^a,b,c^	11.06 ± 0.47 ^a^	0.73 ± 0.02 ^a^	2.50 ± 0.16 ^a^	12.09 ± 0.21 ^a^

Control wine (Ctr), Wine treated by cold stabilization (CS), Wine treated with carboxymethylcellulose (CMC), and Wine treated with metatartaric acid (MetAc). Delphinidin-3-glucoside (D-3-Glc). Cyanidin-3-glucoside (C-3-Glc). Petunidin-3-glucoside (Pet-3-Glc). Peonidin-3-glucoside (Peo-3-Glc). Malvidin-3-glucoside (M-3-Glc). Delphinidin-3-acetylglucoside (D-3-AGlc). Petunidin-3-acetylglucoside (Pet-3-AGlc). Peonidin-3-acetylglucoside (Peo-3-AGlc). Malvidin-3-acetylglucoside (M-3-AGlc). Cyanidin-3-coumaroylglucoside (C-3-CGlc). Petunidin-3-acetylglucoside (Pet-3-CGlc). Malvidin-3-coumaroylglucoside (M-3-CGlc). Columns for each wine followed by the same letter do not present statistically significant differences (Tukey, 5%).

**Table 6:** Chromatic characteristics of white, rosé, and red wine after different tartaric stabilization treatments (mean ± standard deviation).

	Color Intensity (a.u.)	Color _A420 nm_ (a.u.)	Hue	L *	a *	b *	C*	h °	ΔE *
	White wine								
Ctr		0.011 ± 0.001 ^a^	-	98.7 ± 0.1 ^a^	0.23 ± 0.00 ^a^	4.62 ± 0.05 ^a^	4.62 ± 0.05 ^a^	1.52 ± 0.00 ^a^	
CS		0.011 ± 0.003 ^a^	-	98.9 ± 0.0 ^a^	0.02 ± 0.00 ^b^	4.67 ± 0.04 ^a^	4.67 ± 0.04 ^a^	1.57 ± 0.00 ^b^	0.34 ± 0.02 ^a^
CMC		0.012 ± 0.001 ^a^	-	98.7 ± 0.0 ^a^	0.24 ± 0.02 ^a^	4.63 ± 0.03 ^a^	4.64 ± 0.03 ^a^	1.52 ± 0.00 ^a^	0.09 ± 0.01 ^a^
MetAc		0.009 ± 0.000 ^a^	-	98.7 ± 0.1 ^a^	0.21 ± 0.00 ^a^	4.60 ± 0.03 ^a,b^	4.61 ± 0.03 ^a,b^	1.53 ± 0.00 ^a^	0.12 ± 0.01 ^a^
	Rosé wine								
Ctr	2.20 ± 0.07 ^a^		0.802 ± 0.050 ^a^	89.1 ± 2.5 ^a,c^	17.58 ± 0.01 ^a^	5.94 ± 0.27 ^a^	18.56 ± 0.08 ^a^	0.33 ± 0.01 ^a^	
CS	1.32 ± 0.01 ^b^		0.753 ± 0.000 ^a^	94.5 ± 0.1 ^b,c^	8.67 ± 0.08 ^b^	6.12 ± 0.04 ^a^	10.61 ± 0.09 ^b^	0.62 ± 0.00 ^b^	10.55 ± 1.39 ^a^
CMC	2.15 ± 0.01 ^a^		0.775 ± 0.001 ^a^	90.0 ± 1.3 ^c^	17.28 ± 0.01 ^a^	6.14 ± 0.20 ^a^	18.34 ± 0.13 ^a^	0.34 ± 0.01 ^a^	1.06 ± 1.06 ^b^
MetAc	2.24 ± 0.09 ^a^		0.857 ± 0.033 ^a^	90.9 ± 0.2 ^c^	18.12 ± 0.33 ^a^	6.25 ± 0.02 ^a^	19.17 ± 0.30 ^a^	0.33 ± 0.01 ^a^	1.95 ± 2.18 ^b^
	Red wine								
Ctr	6.85 ± 0.02 ^a,c^		0.792 ± 0.001 ^a^	55.7 ± 0.2 ^a^	57.63 ± 0.01 ^a^	5.70 ± 0.30 ^a^	57.91 ± 0.04 ^a^	0.10 ± 0.01 ^a^	
CS	6.30 ± 0.00 ^b^		0.792 ± 0.004 ^a^	59.4 ± 0.0 ^b^	58.63 ± 0.08 ^b^	1.16 ± 0.05 ^b^	58.64 ± 0.08 ^b^	0.02 ± 0.00 ^b^	5.95 ± 0.36 ^a^
CMC	6.84 ± 0.07 ^a,c^		0.791 ± 0.002 ^a^	56.1 ± 0.1 ^a,c^	57.18 ± 0.03 ^c^	5.11 ± 0.02 ^c^	57.41 ± 0.03 ^c^	0.09 ± 0.00 ^a^	0.88 ± 0.22 ^b^
MetAc	6.86 ± 0.01 a		0.792 ± 0.002 ^a^	55.9 ± 0.1 ^a^	57.91 ± 0.01 ^d^	5.92 ± 0.00 ^a^	58.21 ± 0.10 ^a^	0.10 ± 0.00 ^a^	0.45 ± 0.19 ^b^

Control wine (Ctr), Wine treated by cold stabilization (CS), Wine treated with carboxymethylcellulose (CMC), and Wine treated with metatartaric acid (MetAc). L * (lightness), a * (redness), b * (yellowness) coordinates, C * (chroma), h ° (hue-angle), ΔE * (total color difference in relation to control wine). a.u.—absorbance units. Columns for each wine followed by the same letter do not present statistically significant differences (Tukey, 5%).

**Table 7:** Headspace aroma profile of white, rosé, and red wines after different tartaric stabilization treatments (mean ± standard deviation).

					ODT	White				Rosé				Red			
Compounds	ID ^$^	RI Calculate	RI *	Odor Descriptor	µg/L	Ctr	CS	CMC	MetAc	Ctr	CS	CMC	MetAc	Ctr	CS	CMC	MetAc
Ethyl acetate	Std	891	728 ^b^	Fruity, sweet ^b^	7500 ^b^	0.18 ± 0.00 ^a^	0.21 ± 0.00 ^a^	0.18 ± 0.00 ^a^	0.19 ± 0.01 ^a^	0.16 ± 0.01 ^a^	0.17 ± 0.00 ^a^	0.16 ± 0.00 ^a^	0.17 ± 0.01 ^a^	0.05 ± 0.00 ^ab^	0.06 ± 0.00 ^a^	0.06 ± 0.00 ^a^	0.05 ± 0.00 ^b^
3-Methylbutanol acetate	Std	1157	1120 ^a^	Banana, sweet ^a^	30 ^a^	234.8 ± 4.0 ^a^	217.1 ± 14.5 ^a^	214.4 ± 10.2 ^a^	241.2 ± 3.1 ^a^	460.7 ± 3.0 ^b^	503.3 ± 0.5 ^a^	449.8 ± 5.6 ^b^	448.3 ± 15.3 ^b^	16.2 ± 0.2 ^a^	16.4 ± 1.7 ^a^	18.8 ± 0.5 ^a^	19.1 ± 1.0 ^a^
3-Methylbutanol	Std	1193	1206 ^a^	Alcohol, balsamic, burnt^a^	30,000 ^a^	43.7 ± 0.4 ^a^	30.2 ± 0.5 ^b^	41.2 ± 1.7 ^a^	41.4 ± 1.9 ^a^	50.5 ± 3.5 ^a^	41.5 ± 1.3 ^a^	46.1 ± 3.4 ^a^	48.6 ± 1.2 ^a^	153.8 ± 8.9 ^a^	164.4 ± 0.4 ^a^	162.1 ± 9.7 ^a^	151.2 ± 0.5 ^a^
Unknown 1		1216				0.37 ± 0.00 ^a^	0.30 ± 0.03 ^ab^	0.26 ± 0.02 ^b^	0.28 ± 0.03 ^ab^	0.03 ± 0.00 ^a^	0.02 ± 0.00 ^b^	0.02 ± 0.00 ^a,b^	0.02 ± 0.00 ^a,b^				
Ethyl hexanoate	Std	1225	1220 ^a^	Fruit, anise ^a^	5 ^a^	150.1 ± 5.7 ^b^	337.3 ± 9.8 ^a^	146.3 ± 4.6 ^b^	161.8 ± 12.1 ^b^	83.4 ± 1.3 ^b^	132.1 ± 1.6 ^a^	82.9 ± 2.4 ^b^	81.7 ± 0.5 ^b^	33.6 ± 0.00 ^a^	36.9 ± 3.8 ^a^	32.5 ± 1.0 ^a^	28.7 ± 2.1 ^a^
Unknown 2		1253				0.38 ± 0.01 ^a^	0.33 ± 0.03 ^a^	0.34 ± 0.00 ^a^	0.27 ± 0.00 ^b^					0.14 ± 0.01 ^a^	0.11 ± 0.01 ^ab^	0.14 ± 0.01 ^a^	0.10 ± 0.01 ^b^
Hexyl acetate	Std	1260	1262 ^a^	Fruit, citrus ^a^	10 ^a^	13.8 ± 0.1 ^c^	24.8 ± 0.8 ^a^	15.5 ± 0.4 ^b^	16.6 ± 0.5 ^b^	12.0 ± 0.3 ^b^	22.1 ± 0.2 ^a^	11.9 ± 0.6 ^b^	13.0 ± 0.3 ^b^	1.18 ± 0.05 ^b^	0.56 ± 0.03 ^c^	1.45 ± 0.01 ^a^	1.13 ± 0.02 ^b^
γ-Terpinene		1283	1274 ^c^	woody, citrus ^d^	1000 ^d^	1.12 ± 0.01 ^a^	1.04 ± 0.07 ^a,b^	0.81 ± 0.02 ^c^	0.91 ± 0.03 ^bc^	0.07 ± 0.00 ^a^	0.03 ± 0.00 ^b^	0.07 ± 0.00 ^a^	0.06 ± 0.00 ^a^				
Hexanol	Std	1318	1354 ^a^	Floral, sweet, green ^a^	8000 ^a^					0.20 ± 0.00 ^ab^	0.16 ± 0.00 ^b^	0.19 ± 0.02 ^ab^	0.23 ± 0.03 ^a^	0.59 ± 0.04 ^a^	0.43 ± 0.00 ^b^	0.60 ± 0.03 ^a^	0.58 ± 0.04 ^a^
2-Nonanone		1388	1388 ^c^		5.5 ^e^									0.41 ± 0.00 ^b^	0.00 ± 0.00 ^c^	0.48 ± 0.02 ^a^	0.44 ± 0.00 ^b^
Unknown 3		1403				0.59 ± 0.04 ^a^	0.60 ± 0.03 ^a^	0.49 ± 0.02 ^a^	0.54 ± 0.02 ^a^	0.07 ± 0.00 ^a^	0.07 ± 0.00 ^a^	0.04 ± 0.01 ^b^	0.07 ± 0.00 ^a^				
Ethyl octanoate	Std	1419	1414 ^a^	Fruit, waxy ^a^	2 ^a^	738.4 ± 7.6 ^c^	3132.8 ± 25.2 ^a^	901.9 ± 50.0 ^b^	877.8 ± 44.6 ^b^	2035 ± 119 ^b^	2997 ± 283 ^a^	2193 ± 115 ^b^	1933 ± 141 ^b^	148.4 ± 8.0 ^b^	180.0 ± 10.1 ^a^	141.6 ± 1.3 ^b^	131.7 ± 9.2 ^b^
Unknown 4		1465				0.40 ± 0.04 ^a,b^	0.41 ± 0.02 ^a^	0.28 ± 0.01 ^c^	0.31 ± 0.02 ^b,c^								
Linalool	Std	1528	1537 ^a^	Citrus, floral, lavanda ^a^	15 ^a^	19.8 ± 0.6 ^a^	17.1 ± 1.0 ^a^	18.8 ± 1.1 ^a^	19.1 ± 0.7 ^a^	4.58 ± 0.24 ^a^	0.00 ± 0.00 ^b^	3.95 ± 0.34 ^a^	4.42 ± 0.24 ^a^	8.49 ± 0.10 ^b^	9.07 ± 0.32 ^ab^	10.60 ± 0.62 ^a^	9.75 ± 0.67 ^ab^
Unknown 5		1592				2.48 ± 0.10 ^a^	1.88 ± 0.15 ^b^	2.40 ± 0.15 ^a^	2.05 ± 0.10 ^a,b^	0.38 ± 0.03 ^a^	0.00 ± 0.00 ^b^	0.37 ± 0.02 ^a^	0.32 ± 0.02 ^a^				
Ethyl decanoate	Std	1651	1624 ^a^	Fruit, sweet ^a^	200 ^a^	341.8 ± 0.3 ^c^	1479.4 ± 57.5 ^a^	440.1 ± 23.0 ^b^	377.0 ± 29.8 ^bc^	285.7 ± 7.7 ^b^	1735.9 ± 87.0 ^a^	288.7 ± 4.7 ^b^	240.3 ± 9.5 ^c^	72.2 ± 0.0 ^a^	65.1 ± 3.7 ^a,b^	61.1 ± 0.8 ^b,c^	55.8 ± 2.1 ^c^
Unknown 6		1687				0.56 ± 0.05 ^a^	0.54 ± 0.00 ^a^	0.64 ± 0.02 ^a^	0.60 ± 0.04 ^a^								
Unknown 7		1696								0.19 ± 0.01 ^b^	0.24 ± 0.02 ^a^	0.15 ± 0.00 ^c^	0.18 ± 0.01 ^b,c^				
Unknown 8		1709				0.40 ± 0.02 ^a^	0.03 ± 0.00 ^c^	0.40 ± 0.00 ^a^	0.29 ± 0.03 ^b^	0.16 ± 0.00 ^a^	0.09 ± 0.00 ^d^	0.14 ± 0.01 ^b^	0.11 ± 0.00 ^c^				
Unknown 9		1736								0.07 ± 0.00 ^a^	0.02 ± 0.00 ^c^	0.04 ± 0.00 ^b^	0.04 ± 0.00 ^b^				
Ethyl benzeneacetate		1772	1775 ^a^	Fruit ^a^						0.21 ± 0.01 ^b^	0.19 ± 0.01 ^b^	0.19 ± 0.00 ^b^	0.24 ± 0.01 ^a^	0.31 ± 0.02 ^a^	0.30 ± 0.00 ^a^	0.26 ± 0.02 ^a^	0.36 ± 0.03 ^a^
2-Phenylethyl acetate	Std	1797	1883 ^a^	Floral ^a^	250 ^a^	57.5 ± 3.9 ^a^	57.5 ± 0.5 ^a^	57.3 ± 0.2 ^a^	53.3 ± 1.9 ^a^	46.4 ± 1.3 ^a,b^	45.5 ± 0.0 ^ab^	46.9 ± 0.3 ^a^	43.1 ± 1.2 ^b^	9.86 ± 0.30 ^a^	9.53 ± 0.46 ^a^	9.84 ± 0.24 ^a^	10.12 ± 0.89 ^a^
Benzyl alcohol	Std	1836	1869 ^a^	Blackberry, floral, fruit ^a^	20,0000 ^a^									0.93 ± 0.02 ^c^	1.86 ± 0.16 ^a^	1.34 ± 0.03 ^b^	1.23 ± 0.14 ^b,c^
2-Phenylethanol	Std	1870	1910 ^a^	Floral, Honey, spice ^a^	14,000 ^a^	77.6 ± 2.5 ^a^	49.6 ± 4.2 ^c^	64.1 ± 0.2 ^b^	75.2 ± 2.5 ^a,b^	49.0 ± 2.3 ^a^	38.2 ± 3.2 ^b^	52.5 ± 3.4 ^a^	47.6 ± 1.1 ^ab^	202.4 ± 15.6 ^b^	306.0 ± 35.6 ^a^	299.0 ± 13.5 ^a^	386.8 ± 20.7 ^a^
Ethyl dodecanoate	Std	1885	1837 ^a^	Fruity, soap, sweet ^a^	500 ^a^	0.81 ± 0.05 ^b^	3.00 ± 0.01 ^a^	0.66 ± 0.00 ^c^	0.77 ± 0.03 ^b^	0.27 ± 0.02 ^b^	1.39 ± 0.14 ^a^	0.32 ± 0.01 ^b^	0.26 ± 0.02 ^b^	0.52 ± 0.04 ^b^	0.77 ± 0.01 ^a^	0.30 ± 0.03 ^c^	0.25 ± 0.00 ^c^
Hexyl decanoate		1982	1998 ^c^	Fresh green aroma						0.50 ± 0.03 ^b^	3.77 ± 0.39 ^a^	0.65 ± 0.01 ^b^	0.55 ± 0.04 ^b^				
4-Ethylguaiacol	Std	2002	1989 ^b^	Smoke ^b^	150 ^b^									0.07 ± 0.00 ^ab^	0.06 ± 0.00 ^ab^	0.08 ± 0.01 ^a^	0.05 ± 0.00 ^b^
Octanoic acid	Std	2011	2089 ^a^	Cheesy ^a^	10,000 ^a^	95.9 ± 7.2 ^a^	90.1 ± 1.9 ^a^	81.6 ± 1.6 ^a^	89.4 ± 3.3 ^a^	30.3 ± 0.8 ^a^	33.9 ± 3.3 ^a^	32.9 ± 0.7 ^a^	32.7 ± 0.5 ^a^	8.62 ± 0.62 ^a^	8.21 ± 0.70 ^a^	10.03 ± 1.27 ^a^	9.40 ± 0.51 ^a^
Ethyl tetradeconoate		2043	2055 ^c^	Sweet, waxy ^g^	500 ^g^					0.09 ± 0.00 ^b^	0.17 ± 0.01 ^a^	0.09 ± 0.01 ^b^	0.09 ± 0.00 ^b^				
4-Ethylphenol	Std	2123	2142 ^b^	Spicy, phenolic ^b^	400 ^b^									0.09 ± 0.01 ^a^	0.12 ± 0.02 ^a^	0.14 ± 0.00 ^a^	0.08 ± 0.02 ^a^
Decanoic acid	Std	2214		Cheesy, fatty ^a^	15,000 ^a^	3.11 ± 0.14 ^b^	7.02 ± 0.03 ^a^	3.23 ± 0.10 ^b^	3.44 ± 0.08 ^b^	7.36 ± 0.10 ^c^	23.5 ± 0.3 ^a^	8.52 ± 0.24 ^c^	6.87 ± 0.10 ^b^	2.56 ± 0.13 ^a^	2.34 ± 0.36 ^a^	3.18 ± 0.20 ^a^	2.75 ± 0.05 ^a^
Ethyl hexadecanoate		2285	2261 ^c^	Toffee ^f^	1500 ^f^					1.19 ± 0.01 ^c^	2.72 ± 0.04 ^a^	1.28 ± 0.3 ^b^	0.99 ± 0.01 ^d^	1.27 ± 0.06 ^a^	1.26 ± 0.01 ^a^	0.97 ± 0.01 ^b^	1.05 ± 0.06 ^b^
Total esters						1533 ± 1 ^c^	5252 ± 9 ^a^	1728 ± 28 ^b^	1776 ± 88 ^b^	2926 ± 120 ^b^	5444 ± 369 ^a^	2762 ± 165 ^b^	3076 ± 127 ^b^	283.7 ± 3.9 ^a,b^	310.8 ± 16.3 ^a^	248.3 ± 9.3 ^b^	266.9 ± 0.7 ^b^
Total alcohols						121.6 ± 2.0 ^a^	79.8 ± 4.6 ^b^	116.7 ± 4.4 ^a^	105.3 ± 1.5 ^a^	99.7 ± 5.9 ^a^	79.9 ± 1.9 ^b^	94.4 ± 0.0 ^a,b^	98.8 ± 6.8 ^a^	357 ± 6,8 ^b^	472.7 ± 35.3 ^a^	439.8 ± 20.3 ^a^	463.1 ± 23.3 ^a,b^
Total acids						99.0 ± 7.4 ^a^	97.1 ± 1.9 ^a^	92.8 ± 3.3 ^a^	84.9 ± 1.7 ^a^	37.6 ± 0.9 ^b^	57.4 ± 3.6 ^a^	39.6 ± 0.4 ^b^	41.4 ± 0.5 ^b^	11.2 ± 0.5 ^a^	11.5 ± 0.3 ^a^	12.2 ± 0.5 ^a^	13.2 ± 1.5 ^a^
Total terpens						20.9 ± 0.6 ^a^	18.8 ± 1.1 ^a^	20.0 ± 0.7 ^a^	19.6 ± 1.0 ^a^	4.65 ± 0.25 ^a^	0.03 ± 0.00 ^b^	4.48 ± 0.24 ^a^	4.02 ± 0.34 ^a^	8.49 ± 0.10 ^b^	9.07 ± 0.32 ^a,b^	9.75 ± 0.67 ^a,b^	10.6 ± 0.6 ^b^
Total unknowns						5.18 ± 0.06 ^a^	4.09 ± 0.12 ^c^	4.34 ± 0.23 ^a,b^	4.81 ± 0.13 ^b,c^	0.90 ± 0.03 ^a^	0.45 ± 0.01 ^c^	0.74 ± 0.02 ^b^	0.77 ± 0.01 ^b^	0.14 ± 0.01 ^a^	0.11 ± 0.01 ^a,b^	0.10 ± 0.01 ^b^	0.14 ± 0.01 ^a^
Total aroma						1780 ± 11 ^c^	5451 ± 2 ^a^	1990 ± 89 ^b^	1962 ± 29 ^b^	3069 ± 126 ^b^	5582 ± 363 ^a^	2903 ± 166 ^b^	3220 ± 129 ^b^	662 ± 3 ^c^	804 ± 20 ^a^	710 ± 12 ^a,b^	755 ± 25 ^b,c^

Results expressed in absolute area (area/10^5^). ^$^ ID—Identification; std—Standard; * RI (retention index). MW (molecular weight). ODT (olfactory detection threshold). Means within a column followed by the same letter are not significantly different ANOVA and Tuckey post-hoc test (*p* < 0.05). n.d., not detected: Control wine (Ctr), Wine treated by cold stabilization (CS), Wine treated with carboxymethylcellulose (CMC), Wine treated with metatartaric acid (MetAc). Odor descriptor. a [47]; b [48]; c [49]; d [50]; e [51]; f [52]; g [53].

## Data Availability

The original contributions presented in the study are included in the article/Supplementary Materials, further inquiries can be directed to the corresponding authors.

## Supplementary Materials

The following supporting information can be downloaded at https://www.mdpi.com/article/10.3390/foods13172734/s1, Table S1. Eigenvalues obtained after Principal Component Analysis of white wines volatilome (after logarithmic transformation of the original values to standardize the variance) before and after application of tartaric stabilization techniques. Table S2. Factor Loadings obtained after Principal Component Analysis and Varimax Rotation of white wine volatilome (after logarithmic transformation of the original values to standardize the variance) before and after the application of tartaric stabilization techniques. Marked loadings are >0.700. Table S3. Factor Scores obtained after Principal Component Analysis and Varimax Rotation of white wine volatilome (after logarithmic transformation of the original values to standardize the variance) before and after the application of tartaric stabilization techniques. Table S4. Eigenvalues obtained after Principal Component Analysis of rosé wines volatilome (after logarithmic transformation of the original values to standardize the variance) before and after application of tartaric stabilization techniques. Table S5. Factor Loadings obtained after Principal Component Analysis and Varimax Rotation of rosé wines volatilome (after logarithmic transformation of the original values to standardize the variance) before and after the application of tartaric stabilization techniques. Marked loadings are >0.700. Table S6. Factor Scores obtained after Principal Component Analysis and Varimax Rotation of rosé wines volatilome (after logarithmic transformation of the original values to standardize the variance) before and after the application of tartaric stabilization techniques. Table S7. Eigenvalues obtained after Principal Component Analysis of red wine volatilome (after logarithmic transformation of the original values to standardize the variance) before and after the application of tartaric stabilization techniques. Table S8. Factor Loadings obtained after Principal Component Analysis and Varimax Rotation of red wine volatilome (after logarithmic transformation of the original values to standardize the variance) before and after the application of tartaric stabilization techniques. Marked loadings are >0.700. Table S9. Factor Scores obtained after Principal Component Analysis and Varimax Rotation of rosé wines volatilome (after logarithmic transformation of the original values to standardize the variance) before and after the application of tartaric stabilization techniques.

## References

[1] Lasanta C., Gomez J. (2012). Tartrate stabilization of wines. Trends Food Sci..

[2] Bott E.W. (1988). A new tartrate stabilization technology successfully in operation. Aust. Grapegrow. Winemak..

[3] Gerbaud V., Gabas N., Blouin J., Crachereau J.C. (2010). Study of wine tartaric acid salt stabilization by addition of carboxymethylcellulose (CMC): Comparison with the “Protective colloids” effect. J. Int. Sci. Vigne Vin..

[4] Martínez-Pérez M.P., Bautista-Ortín A.B., Durant V., Gómez-Plaza E. (2020). Evaluating alternatives to cold stabilization in wineries: The use of carboxymethyl cellulose, potassium polyaspartate, electrodialysis and ion exchange resins. Foods.

[5] OIV (Organisation International de la Vigne et du Vin) (2023). International Code of Oenological Practices.

[6] Blouin J. (1982). Les techniques de stabilisation tartrique des vins par le froid. Connaiss. Vigne Vin..

[7] Boulton R.B., Singleton V.L., Bisson L.F., Kunkee R.E. (1995). Principles and Practices of Winemaking.

[8] Ribéreau-Gayon P., Glories Y., Maujean A., Dubourdieu D. (2006). Handbook of Enology. The Chemistry of Wine Stabilization and Treatments.

[9] Vernhet A., Dupre K., Boulange-Petermann L., Cheynier V., Pellerin P., Moutounet M. (1999). Composition of tartrate precipitates deposited on stainless steel tanks during the cold stabilization of wines. Part I. White wines. Am. J. Enol. Vitic..

[10] Bories A., Sire Y., Bouissou D., Goulesque S., Moutounet M., Bonneaud D., Lutin F. (2011). Environmental impacts of tartaric stabilisation processes for wines using electrodialysis and cold treatment. S. Afr. J. Enol. Vitic..

[11] Langlois M. (1992). La stabilisation tartrique des vins en continu contact par le système Ingevins. Rev. Des OEnologues.

[12] Gómez-Benítez J., Palacios V.M., Gorostiaga P., Veas R., Pérez L. (2003). Comparison of electrodialysis and cold treatment on an industrial scale for tartrate stabilization of sherry wines. J. Food Eng..

[13] Low L.L., O’Neill B., Ford C., Godden J., Gishen M., Colby C. (2008). Economic evaluation of alternative technologies for tartrate stabilisation of wines. Int. J. Food Sci. Technol..

[14] Vogdt J.E., Schleenstein G. Wastewater characteristics from Chilean wineries. Proceedings of the 4th International Specialized Conference on Sustainable Viticulture: Winery Wastes and Ecologic Impact Management.

[15] Ding H., Hou R., Li Y., Zhang B., Zhao B., Liu K. (2020). Effect of different carboxymethyl cellulose structure parameters on tartrates stability of red wine: Viscosity and degree of substitution. Food Addit. Contam. Part A.

[16] Crachereau J.C., Gabas N., Blouin J., Hébrard B., Maujean A. (2001). Stabilisation tartrique des vins par la carboxyméthylcellulose. Bull. De L’OIV.

[17] Guise R., Filipe-Ribeiro L., Nascimento D., Bessa O., Nunes F.M., Cosme F. (2014). Comparison between different types of carboxylmethylcellulose and other oenological additives used for white wine tartaric stabilization. Food Chem..

[18] Stojanovic Z., Jeremic K., Jovanovic S., Lechnerb M.D. (2005). A comparison of some methods for the determination of the degree of substitution of carboxymethylcellulose starch. Starch/Stärke.

[19] Rodriguez-Clemente R., Correa-Gorospe I., De Castro J.J. (1990). A new method for the stabilization of wines with respect to the potassium bitartrate precipitation. Am. J. Enol. Vitic.

[20] Greeff A., Robillard B., Toit W. (2012). Short- and long-term efficiency of carboxymethylcellulose (CMC) to prevent crystal formation in South African wine. Food Addit. Contam. Part A.

[21] Lubbers S., Léger B., Charpentier C., Feuillat M. (1993). Effet colloïde-protecteur d’extraits de parois de levure sur la stabilité tartrique d’une solution hydro alcoolique modèle. J. Int. Sci. Vigne Vin.

[22] Salagoїty M.H., Guyon F., René L., Gaillard L., Lagrèze C., Domec A., Baudouin M., Médina B. (2011). Quantification method and organoleptic impact of added carboxymethylcellulose to dry white wine. R. Soc. Chem..

[23] Bowyer P., Gouty C., Moine V., Marsh R., Battaglene T. (2010). CMC a new potassium bitartrate stabilisation tool. Aust. N. Z. Grapegrow. Winemak..

[24] Moutounet M., Bouissou D., Escudier J.L. (2010). Effets de traitement de stabilisation tartrique de vins rouges par une gomme de cellulose. Infowine.

[25] Claus H., Tenzer S., Sobe M., Schlander M., König H., Fröhlich J. (2014). Effect of carboxymethyl cellulose on tartrate salt, protein and colour stability of red wine. Aust. J. Grape Wine Res..

[26] Sommer S., Dickescheid C., Harbertson J.F., Fischer U., Cohen S.D. (2016). Rationale for haze formation after carboxymethyl cellulose (CMC) addition to red wine. J. Agric. Food Chem..

[27] Filipe-Ribeiro L., Milheiro J., Guise R., Vilamarim R., Fraga J.B., Martins-Gomes C., Nunes F.M., Cosme F. (2021). Efficiency of carboxymethylcellulose in red wine tartaric stability: Effect on wine phenolic composition, chromatic characteristics and colouring matter stability. Food Chem..

[28] Pittari E., Catarino S., Andrade M.C., Ricardo-da-Silva J.M. (2018). Preliminary results on tartaric stabilization of red wine by adding different carboxymethylcelluloses. Cienc. Tec. Vitivinic.

[29] Sprenger S., Hirn S., Dietrich H., Will F. (2015). Metatartaric acid: Physicochemical characterization and analytical detection in wines and grape juices. Eur. Food Res. Technol..

[30] Cozzolino R., Martignetti A., Pellicano M.P., Stocchero M., Cefola M., Pace B., De Giulio B. (2016). Characterisation of volatile profile and sensory analysis of fresh-cut “Radicchio di Chioggia” stored in air or modified atmosphere. Food Chem..

[31] Peterson A., Cholet C., Geny L., Darriet P., Landais Y., Pons A. (2020). Identification and analysis of new α- and β-hydroxy ketones related to the formation of 3-methyl-2,4-nonanedione in musts and red wines. Food Chem..

[32] Slaghenaufi D., Ugliano M. (2018). Norisoprenoids, sesquiterpenes and terpenoids content of valpolicella wines during aging: Investigating aroma potential in relationship to evolution of tobacco and balsamic aroma in aged wine. Front. Chem..

[33] Pascual G.A., Serra I., Calderón-Orellana A., Laurie V.F., Lopéz M.D. (2017). Changes in concentration of volatile compounds in response to defoliation of Muscat of Alexandria grapevines grown under a traditional farming system. Chil. J. Agric. Res..

[34] Bueno M., Zapata J., Ferreira V. (2014). Simultaneous determination of free and bonded forms of odor-active carbonyls in wine using a headspace solid phase microextraction strategy. J. Chromatogr. A.

[35] Cameira-dos-Santos P., Pereira O.M., Simões F.J., Pinho T.M.N. (2000). Ensaios de estabilização tartárica em vinhos portugueses: Estudo comparativo da eletrodiálise e de um método tradicional. Cienc. Tec. Vitivinic.

[36] Bosso A., Salmaso D., Faveri E., Guaita M., Franceshi D. (2010). The use of carboxymethylcellulose for the tartaric stabilization of white wines, in comparison with other oenological additives. Vitis.

[37] Bullio C. (2002). TartarCheck. User’s Handbook.

[38] OIV (2023). Récueil de Méthodes Internationeiles d’Analyse des Vins et des Moûts.

[39] Vidal M., Blouin J. (1978). Dosage colorimétrique rapide de l’acid tartrique dans les mouts et les vins (Méthode Rebelein modifié). Rev. Française d’OEnologie.

[40] Kramling T.E., Singleton V.L. (1969). An estimate of the nonflavonoid phenols in wines. Am. J. Enol. Vitic..

[41] Somers T.C., Evans M.E. (1977). Spectral evaluation of young red wines: Anthocyanin equilibria, total phenolics, free and molecular SO_2_, “Chemical age”. J. Sci. Food Agric..

[42] Singleton V.L., Kramling T.E. (1976). Browning of white wines and accelerated test for browning capacity. Am. J. Enol. Vitic..

[43] Filipe-Ribeiro L., Milheiro J., Matos C.C., Cosme F., Nunes F.M. (2017). Reduction of 4-ethylphenol and 4-ethylguaiacol in red wine by activated carbons with different physicochemical characteristics: Impact on wine quality. Food Chem..

[44] Manly B. (2004). Multivariate Statistical Methods: A Primer.

[45] Bajul A., Gerbaud V., Teychene S., Devatine A., Bajul G. (2017). Effect of carboxymethylcellulose on potassium bitartrate crystallization on model solution and white wine. J. Cryst. Growth.

[46] Pardo-García A.I., De La Hoz K.S., Zalacain A., Alonso G.L., Salinas M.R. (2014). Effect of vine foliar treatments on the varietal aroma of Monastrell wines. Food Chem..

[47] Perestrelo R., Silva C., Câmara J.S. (2019). Madeira Wine Volatile Profile. A Platform to Establish Madeira Wine Aroma Descriptors. Molecules.

[48] Filipe-Ribeiro L., Milheiro J., Matos C.C., Cosme F., Nunes F.M. (2017). Data on changes in red wine phenolic compounds, headspace aroma compounds and sensory profile after treatment of red wines with activated carbons with different physicochemical characteristics. Data Brief.

[49] National Institute of Standards and Technology U.S. Department of Commerce. https://www.nist.gov.

[50] Niu Y., Wang P., Xiao Q., Xiao Z., Mao H., Zhang J. (2020). Characterization of Odor-Active Volatiles and Odor Contribution Based on Binary Interaction Effects in Mango and Vodka Cocktail. Molecules.

[51] Cometto-Muñiz J.E., Abraham M.H. (2009). Olfactory psychometric functions for homologous 2-ketones. Behav. Brain Res..

[52] Hong J., Wang J., Zhang C., Zhao Z., Tian W., Wu Y., Chen H., Zhao D., Sun J. (2021). Unraveling variation on the profile aroma compounds of strong aroma type of Baijiu in different regions by molecular matrix analysis and olfactory analysis. RSC Adv..

[53] Guan Q., Meng L.-J., Mei Z., Liu Q., Chai L.-J., Zhong X.-Z., Zheng L., Liu G., Wang S., Shen C. (2022). Volatile Compound Abundance Correlations Provide a New Insight into Odor Balances in Sauce-Aroma Baijiu. Foods.

[54] Sánchez-Palomo E., Delgado J.A., Ferrer M.A., Viñas M.A.G. (2019). The aroma of La Mancha Chelva wines: Chemical and sensory characterization. Food Res. Int..

[55] Rice S., Lutt N., Koziel J.A., Dharmadhikari M., Fennell A. (2018). Determination of selected aromas in marquette and frontenac wine using headspace-SPME coupled with GC-MS and simultaneous olfactometry. Separations.

[56] Sanchez-Palomo E., Díaz-Maroto M.C., Viñas M.A.G., Soriano-Pérez A., Pérez-Coello M.S. (2007). Aroma profile of wines from Albillo and Muscat grape varieties at different stages of ripening. Food Control.

[57] Niu Y., Zhu Q., Xiao Z. (2020). Characterization of perceptual interactions among ester aroma compounds found in Chinese Moutai Baijiu by gas chromatography-olfactometry, odor Intensity, olfactory threshold and odor activity value. Food Res. Int..

[58] Etschmann M.M.W., Kötter P., Hauf J., Bluemke W., Entian K.D., Schrader J. (2008). Production of the aroma chemicals 3-(methylthio)-1-propanol and 3-(methylthio)-propylacetate with yeasts. Appl. Microbiol. Biotechnol..

[59] Genovese A., Gambuti A., Piombino P., Moio L. (2007). Sensory properties and aroma compounds of sweet Fiano wine. Food Chem..

[60] Chatonnet P., Dubourdieu D., Boidron J.-N., Lavigne V. (1993). Synthesis of volatile phenols by *Saccharomyces cerevisiae* in wines. J. Sci. Food Agric..

[61] Francis I.L., Newton J.L. (2005). Determining wine aroma from compositional data. Aust. J. Grape Wine Res..

[62] Gómez-Plaza E., Gil-Muñoz R., Hernández-Jiménez A., López-Roca J.M., Ortega-Regules A., Martínez-Cutillas A. (2008). Studies on the anthocyanin profile of *Vitis Vinifera* intraspecific hybrids (Monastrell × Cabernet Sauvignon). Eur. Food Res. Technol..

[63] Lorenzo C., Pardo F., Zalacain A., Alonso G.L., Salinas M.R. (2008). Differentiation of co-winemaking wines by their aroma composition. Eur. Food Res. Technol..

[64] Kotseridis Y., Baumes R. (2000). Identification of impact odorants in Bordeaux red grape juice, in the commercial yeast used for its fermentation, and in the produced wine. J. Agric. Food Chem..

[65] Vilanova M., Genisheva Z., Masa A., Oliveira J.M. (2010). Correlation between volatile composition and sensory properties in Spanish Albariño wines. Microchem. J..

[66] Wang X., Tao Y.-S., Wu Y., An R., Yue Z.-Y. (2017). Aroma compounds and characteristics of noble-rot wines of Chardonnay grapes artificially botrytized in the vineyard. Food Chem..

[67] Gil-Muñoz R., Jiménez-Martínez M.D., Bautista-Ortín A.B., Gómez-Plaza E. (2019). Effect of the use of purified grape pomace as a fining agent on the volatile composition of monastrell wines. Molecules.

[68] Pedroza M.A., Carmona M., Pardo F., Salinas M.R., Zalacain A. (2012). Waste grape skins thermal dehydration: Potential release of colour, phenolic and aroma compounds into wine. CYTA J. Food.

[69] Chatonnet P., Dubourdieu D., Boidron J.N., Poins M. (1992). The origin of ethylphenols in wines. J. Sci. Food Agric..

[70] Gonzalez-Viñas M.A., Perez-Coello M.S., Salvador M.D., Cabezudo M.D., Martin-Alvarez P.J. (1996). Changes in gas-chromatographic volatiles of young Airen wines during bottle storage. Food Chem..

[71] Sánchez-Palomo E., Alonso-Villegas R., González-Viñas M.A. (2015). Characterisation of free and glycosidically bound aroma compounds of la Mancha Verdejo white wines. Food Chem..

[72] Xia N., Cheng H., Yao X., Pan Q., Meng N., Yu Q. (2022). Effect of cold stabilization duration on organic acids and aroma compounds during *Vitis vinifera* L. cv. Riesling wine bottle storage. Foods.

[73] Pozo-Bayón M.Á., Reineccius G. (2009). Interactions Between Wine Matrix Macro-Components and Aroma Compounds. Wine Chemistry and Biochemistry.

[74] Cameleyre M., Lytra G., Barbe J.-C. (2018). Static Headspace Analysis Using Low-Pressure Gas Chromatography and Mass Spectrometry, Application to Determining Multiple Partition Coefficients: A Practical T9ool for Understanding Red Wine Fruity Volatile Perception and the Sensory Impact of Higher Alcohols. Anal. Chem..

[75] Dufour C., Bayonove C.L. (1999). Influence of Wine Structurally Different Polysaccharides on the Volatility of Aroma Substances in a Model System. J. Agric. Food Chem..

[76] Muñoz-González C., Martín-Álvarez P.J., Moreno-Arribas M.V., Pozo-Bayón M.Á. (2014). Impact of the nonvolatile wine matrix composition on the in vivo aroma release from wines. J. Agric. Food Chem..

[77] Pittari E., Moio L., Piombino P. (2021). Interactions between Polyphenols and Volatile Compounds in 781 Wine: A Literature Review on Physicochemical and Sensory Insights. Appl. Sci..

[78] Dufour C., Bayonove C.L. (1999). Interactions between Wine Polyphenols and Aroma Substances. An Insight at the Molecular Level. J. Agric. Food Chem..

