# Supplementary material for: Wine Volatilome as Affected by Tartaric Stabilization Treatments: Cold Stabilization, Carboxymethylcellulose and Metatartaric Acid
Source: Foods. 2024 Aug 28;13(17):2734. doi: 10.3390/foods13172734 (PMC11394989; doi:10.3390/foods13172734)
Supplement: Supplementary file 1 [file foods-13-02734-s001.zip › foods-3058377-supplementary.pdf]

Supplementary Material

Wine Volatilome as Affected by Tartaric Stabilization Treatments: Cold Stabilization, Carboxymethylcellulose and Metatartaric Acid

Fernanda Cosme[1*], Rui Oliveira[2], Luís Filipe-Ribeiro[2] and Fernando M. Nunes[3,*]

[1] CQ-VR, Chemistry Research Centre – Vila Real, Food and Wine Chemistry Lab, University of Trás-os-Montes and Alto Douro, Biology and Environment Department, 5000 801 Vila Real, Portugal.; fcosme@utad.pt
[2] CQ-VR, Chemistry Research Centre – Vila Real, Food and Wine Chemistry Lab, University of Trás-os-Montes and Alto Douro, 5000 801 Vila Real, Portugal; ruioliveirapf@gmail.com; fmota @utad.pt
[3] CQ-VR, Chemistry Research Centre – Vila Real, Food and Wine Chemistry Lab, University of Trás-os-Montes and Alto Douro, Chemistry Department, 5000-801 Vila Real, Portugal. fnunes@utad.pt
* Correspondence: fcosme@utad.pt; fnunes@utad.pt

Table S1. Eigenvalues obtained after Principal Component Analysis of white wines volatilome (after logarithmizing the original values to standardize the variance) before and after application of tartaric stabilisation techniques

Value	Eigenvalue	% Total variance	Cumulative Eigenvalue	Cumulative %
1	12,99836	59,08343	12,99836	59,08343
2	5,02164	22,82566	18,02000	81,90909
3	2,32608	10,57308	20,34608	92,48217

Table S2. Factor Loadings obtained after Principal Component Analysis and Varimax Rotation of white wines volatilome (after logarithmizing the original values to standardize the variance) before and after application of tartaric stabilisation techniques. Marked loadings are >0.700

Variable	Factor 1	Factor 2	Factor 3
Ethyl acetate	0,90256	0,125495	-0,269978
3-Methylbutanol acetate	-0,46618	0,407857	-0,718485
3-Methylbutanol	-0,97602	-0,079823	-0,114318
Unknown 1	-0,20861	0,935099	0,157080
Ethyl hexanoate	0,97144	0,213843	-0,016162
Unknown 2	-0,19314	0,398421	0,742943
Hexyl acetate	0,99236	-0,014456	-0,085050
γ-Terpinene	0,13006	0,972648	0,047809
Unknown 3	0,39017	0,827487	0,035818
Ethyl octanoate	0,98867	0,083405	0,075136
Unknown 4	0,42835	0,828408	0,104527
Linalool	-0,88977	0,184092	-0,071025
Unknown 5	-0,80682	-0,018082	0,518063
Ethyl decanoate	0,97674	0,068904	0,167315
Unknown 6	-0,47966	-0,752364	-0,037698
Unknown 8	-0,97971	-0,193401	0,009896
2-Phenylethyl acetate	0,13240	0,239730	0,877730
2-Phenylethanol	-0,92448	0,182853	-0,315067
Ethyl dodecanote	0,94754	0,305623	0,052901
Octanoic acid	-0,07934	0,934584	-0,061670
Decanoic acid	0,97904	0,175064	0,044415
Total	0,98357	0,123485	0,089439
Expl.Var	12,61280	5,337628	2,395647
Prp.Totl	0,57331	0,242619	0,108893

Table S3. Factor Scores obtained after Principal Component Analysis and Varimax Rotation of white wines volatilome (after logarithmizing the original values to standardize the variance) before and after application of tartaric stabilisation techniques

Case	Factor 1	Factor 2	Factor 3
Ctr1	-0,804487	0,93623	0,04868
Ctr2	-0,932674	1,46760	0,82323
CS1	1,448810	0,65992	-0,12442
CS2	1,688094	0,02561	0,49658
MetAc1	-0,435497	-0,05461	-1,02669
MetAc2	-0,181877	-0,46795	-1,89070
CMC1	-0,339133	-1,19051	0,95709
CMC2	-0,443236	-1,37630	0,71623

Table S4. Eigenvalues obtained after Principal Component Analysis of rosé wines volatilome (after logarithmizing the original values to standardize the variance) before and after application of tartaric stabilisation techniques

Value	Eigenvalue	% Total variance	Cumulative Eigenvalue	Cumulative %
1	18,62342	71,62855	18,62342	71,62855
2	3,21898	12,38068	21,84240	84,00923
3	2,34991	9,03810	24,19230	93,04732

Table S5. Factor Loadings obtained after Principal Component Analysis and Varimax Rotation of rosé wines volatilome (after logarithmizing the original values to standardize the variance) before and after application of tartaric stabilisation techniques. Marked loadings are >0.700

Variable	Factor 1	Factor 2	Factor 3
Ethyl acetate	0,34473	-0,657022	0,337486
3-Methylbutanol acetate	0,95111	0,029694	0,063866
3-Methylbutanol	-0,70899	-0,188002	-0,495036
Unknown 1	-0,38789	0,203598	-0,809512
Ethyl hexanoate	0,97714	0,029702	0,198294
Hexyl acetate	0,96090	-0,112751	0,221246
γ-Terpinene	-0,90934	0,232393	-0,275170
Hexanol	-0,76325	-0,543970	-0,140488
Unknown 3	0,52494	-0,536462	-0,621401
Ethyl octanoate	0,89185	0,184397	0,290870
Linalool	-0,97286	-0,005696	-0,225290
Unknwon 5	-0,97338	0,012643	-0,222446
Ethyl decanoate	0,97602	0,078112	0,198386
Unknwon 7	0,93401	-0,248610	-0,219824
Unknwon 8	-0,70369	0,408492	-0,530995
Unknown 9	-0,69892	0,116078	-0,685966

Ethyl benzeneacetate	-0,36420	-0,827855	-0,187177
2-Phenylethyl acetate	0,06219	0,963449	-0,215384
2-Phenylethanol	-0,93317	0,219884	0,005278
Ethyl dodecanoate	0,96100	0,100933	0,250887
Hexyl decanoate	0,95021	0,035745	0,306799
Octanoico acid	0,26874	-0,059710	0,781936
Ethyl tetradeconoate	0,98961	0,000407	0,092570
Decanoic acid	0,95005	0,142025	0,275289
Ethyl hexadecanoate	0,94748	0,235449	0,209459
Total	0,95385	0,107690	0,245949
Expl.Var	17,33314	3,207259	3,651901
Prp.Totl	0,66666	0,123356	0,140458

Table S6. Factor Scores obtained after Principal Component Analysis and Varimax Rotation of rosé wines volatilome (after logarithmizing the original values to standardize the variance) before and after application of tartaric stabilisation techniques

Case	Factor 1	Factor 2	Factor 3
Ctr1	-0,190357	0,58421	-1,63547
Ctr2	-0,256660	0,01668	-1,35899
CS1	1,428281	0,05419	0,71878
CS2	1,730134	-0,05704	-0,01274
MetAc1	-0,599945	-1,06790	-0,05567
MetAc2	-0,643190	-1,74745	0,60218
CMC1	-0,814792	1,10895	1,06934
CMC2	-0,653471	1,10836	0,67256

Table S7. Eigenvalues obtained after Principal Component Analysis of red wines volatilome (after logarithmizing the original values to standardize the variance) before and after application of tartaric stabilisation techniques

Value	Eigenvalue	% Total variance	Cumulative Eigenvalue	Cumulative %
1	8,770433	39,86561	8,77043	39,86561
2	5,980688	27,18495	14,75112	67,05055
3	4,442138	20,19154	19,19326	87,24209
4	1,175414	5,34279	20,36867	92,58488

Table S8. Factor Loadings obtained after Principal Component Analysis and Varimax Rotation of red wines volatilome (after logarithmizing the original values to standardize the variance) before and after application of tartaric stabilisation techniques. Marked loadings are >0.700

Variable	Factor 1	Factor 2	Factor 3	Factor 4
Ethyl acetate	0,020471	-0,448744	0,816540	0,236883
3-Methylbutanol acetate	0,941688	0,010765	-0,151946	0,277883
3-Methylbutanol	-0,078270	-0,651896	0,489785	-0,043029
Ethyl hexanoate	-0,753414	-0,316854	0,434003	0,250555
Unknwon 2	-0,211761	0,301225	0,901955	-0,112533
Hexyl acetate	0,636392	0,706710	0,289855	-0,099035
Hexanol	0,547243	0,788173	0,179434	0,052171
2-Nonanone	0,561425	0,809365	0,071967	-0,144921
Ethyl octanoate	-0,751338	-0,563755	0,180646	0,250243
Linalool	0,880203	-0,331951	0,263756	-0,188494
Ethyl decanoate	-0,807004	0,283293	0,463372	-0,165093
Ethyl Benzeneacetate	0,025118	0,162851	-0,883256	0,046512
2-Phenylethyl acetate	0,232961	0,186061	-0,107520	-0,931894
Benzyl alcohol	-0,003213	-0,989213	-0,001182	0,051966
2-Phenylethanol	0,523688	-0,821285	-0,048964	0,078093
Ethyl dodecanoate	-0,871962	-0,358702	0,185925	0,113726
4-Ethylguaiacol	0,060852	0,172476	0,943277	0,150254
Octanoico acid	0,841262	0,057698	0,211557	-0,254748
4 Ethylphenol	0,113401	-0,489850	0,779397	-0,019499
Decanoic acid	0,022939	-0,806106	0,376166	0,111599
Ethyl hexadecanoate	-0,925044	0,049563	-0,177325	0,200351
Total	0,094693	-0,973623	0,168706	0,108393
Expl.Var	7,194948	6,817675	4,941966	1,414084
Prp.Totl	0,327043	0,309894	0,224635	0,064277

Table S9. Factor Scores obtained after Principal Component Analysis and Varimax Rotation of rosé wines volatilome (after logarithmizing the original values to standardize the variance) before and after application of tartaric stabilisation techniques

Case	Factor 1	Factor 2	Factor 3	Factor 4
Ctr1	-0,86320	1,36345	0,32383	0,40107
Ctr2	-0,92169	1,12945	0,15392	-0,66409
CS1	-0,42298	-1,41675	-0,25653	0,84687
CS2	-1,36818	-1,22607	0,03879	-0,37134
MetAc1	0,75512	0,46259	-1,48970	1,43120
MetAc2	0,84209	-0,09448	-1,22430	-1,82089
CMC1	1,23161	-0,34170	1,42060	-0,19721
CMC2	0,74724	0,12350	1,03339	0,37440
